# In vitro effect of visfatin on endocrine functions of the porcine corpus luteum

**DOI:** 10.1038/s41598-024-65102-4

**Published:** 2024-06-26

**Authors:** Ewa Mlyczyńska, Edyta Rytelewska, Ewa Zaobidna, Natalia Respekta-Długosz, Grzegorz Kopij, Kamil Dobrzyń, Marta Kieżun, Nina Smolińska, Tadeusz Kamiński, Agnieszka Rak

**Affiliations:** 1 Laboratory of Physiology and Toxicology of Reproduction, Institute of Zoology and Biomedical Research, Jagiellonian University in Krakow, Krakow, Poland; 2 Doctoral School of Exact and Natural Sciences, Jagiellonian University in Krakow, Krakow, Poland; 3 Department of Animal Anatomy and Physiology, Faculty of Biology and Biotechnology, University of Warmia and Mazury in Olsztyn, Olsztyn-Kortowo, Poland; 4 Department of Biochemistry, Faculty of Biology and Biotechnology, University of Warmia and Mazury in Olsztyn, Olsztyn-Kortowo, Poland; 5 Department of Zoology, Faculty of Biology and Biotechnology, University of Warmia and Mazury in Olsztyn, Olsztyn-Kortowo, Poland

**Keywords:** Endocrine system and metabolic diseases, Cell biology

## Abstract

Previously, we demonstrated the expression of visfatin in porcine reproductive tissues and its effect on pituitary endocrinology. The objective of this study was to examine the visfatin effect on the secretion of steroid (P_4_, E_2_) and prostaglandin (PGE_2_, PGF_2α_), the mRNA and protein abundance of steroidogenic markers (STAR, CYP11A1, HSD3B, CYP19A1), prostaglandin receptors (PTGER2, PTGFR), insulin receptor (INSR), and activity of kinases (MAPK/ERK1/2, AKT, AMPK) in the porcine corpus luteum. We noted that the visfatin effect strongly depends on the phase of the estrous cycle: on days 2–3 and 14–16 it reduced P_4_, while on days 10–12 it stimulated P_4_. Visfatin increased secretion of E_2_ on days 2–3, PGE_2_ on days 2–3 and 10–12, reduced PGF_2α_ release on days 14–16, as well as stimulated the expression of steroidogenic markers on days 10–12 of the estrous cycle. Moreover, visfatin elevated PTGER mRNA expression and decreased its protein level, while we noted the opposite changes for PTGFR. Additionally, visfatin activated ERK1/2, AKT, and AMPK, while reduced INSR phosphorylation. Interestingly, after inhibition of INSR and signalling pathways visfatin action was abolished. These findings suggest a regulatory role of visfatin in the porcine corpus luteum.

## Introduction

The corpus luteum (CL) is a transient gland that is formed in the mammalian ovary, and its proper functioning determines the maintenance of pregnancy as well as the cyclicity of the ovary. The CL performs its function mainly by secreting steroid hormones. The most important of them, progesterone (P_4_), prepares the uterine wall for embryo implantation and prevents its contractions and further rejection of the fetus^1^. Estradiol (E_2_), although secreted in smaller amounts, affects the generation of the CL by the formation of blood vessels^2^. In the process of steroidogenesis, in addition to the steroidogenic acute regulatory protein (STAR), which transports cholesterol from the outer to the inner mitochondrial membrane, two main types of enzymes are involved: cytochromes P450 and steroid oxidoreductases. Cytochrome P450 family 11 subfamily A member 1 (CYP11A1) and hydroxy-delta-5-steroid dehydrogenase (HSD3B) are enzymes involved in the synthesis of P_4_, while cytochrome P450 family 19 subfamily A member 1 (CYP19A1) is responsible for the conversion of androgens to estrogens, including E_2_^3^. In addition, the CL synthesizes and secretes prostaglandins such as prostaglandin E_2_ (PGE_2_) and prostaglandin F_2α_ (PGF_2α_), which act through specific receptors: PTGER isoforms and PTGFR and control luteinization and luteolysis, respectively^4^. Together, the aforementioned hormones create a unique environment for the proper functioning of the CL. Recent studies also point to other important factors connected with the development, lifespan, and regression of the CL, including adipokines^5^. Most of them have a luteotropic effect: apelin stimulates P_4_ secretion by increasing HSD3B expression^6^, and vaspin upregulates P_4_ and E_2_ synthesis and increases the ratio of PGE_2_ to PGF_2α_ secretion in the porcine CL^7^. In contrast, adiponectin causes a decrease in P_4_ secretion by porcine luteal cells isolated in the mid-luteal phase of the cycle^8^. Disturbances in the luteal endocrine function lead to numerous negative consequences, primarily luteal phase deficiency, defined as decreased production of P_4_, both in quantity and duration. Luteal phase dysfunction may result in premature regression of the gland and subsequent transition to an infertile cycle^9^. Understanding the mechanism of steroidogenesis during the development and regression of the CL is crucial for assessing the proper physiology and pathophysiology of reproductive cycles.

Visfatin exists in two functional isoforms^10^. An intracellular form called nicotinamide phosphoribosyltransferase (iNAMPT) acts as the rate-limiting enzyme in the mammalian NAD biosynthetic pathway. It is also worth noting that visfatin can undergo dimerization, which is necessary for its enzymatic activity^11^. A competitive pharmacological blocker of NAMPT activity is FK866, which binds to the active site in NAMPT dimers; therefore, it is a useful agent for studying the enzymatic action of visfatin^12^. In turn, the extracellular form of visfatin (eNAMPT) is considered an adipokine. It is secreted, acts through a specific receptor or as an ectoenzyme, and has a pleiotropic effect in the body^10^. The insulin-mimetic effect of visfatin is achieved through increasing glucose uptake in myocytes and adipocytes, inhibiting hepatic glucose release, and stimulating the accumulation of triglycerides^13^. Visfatin may activate the insulin receptor (INSR) by binding at a site other than insulin (INS) and thus act in target cells including pancreatic cells^14^, osteoblasts^15^, and renal mesangial cells^16^. More recently, several other putative visfatin cell surface receptors have been identified—for example, C–C chemokine receptor type 5 (CCR5) and Toll-like receptor 4 (TLR4)—with a dissociation constant (Kd) in the nanomolar range, indicating high affinity^17^.

The presence of visfatin at the mRNA and protein levels has been found in the ovary of women^18^, mice^19,20^, chickens^21^, cattle^22,23^, and pigs^24,25^. Visfatin regulates steroidogenesis^18,21–23^, improves the maturation of oocytes in mice^26^ and women^27^, and inhibits apoptosis of granulosa cells (Gc)^20^. Nevertheless, there is limited knowledge regarding the contribution of visfatin to the functionality of luteal cells. So far, Tharke et al.^23^ observed that in water buffalo visfatin stimulates P_4_ secretion by luteal cells; however, the effect of visfatin on ovarian steroidogenesis is ambiguous. In chicken Gc, visfatin reduces P_4_ secretion^21^, while in human Gc it increases the release of both P_4_ and E_2_^18^, suggesting an important, species-specific role of visfatin in ovarian steroidogenesis.

Our previous studies have shown that visfatin is present in porcine luteal cells and its protein level is highest in the mid-luteal phase^24^, which may indicate its participation in maintaining the secretory function of the CL. Considering these observations as well as the evidence for the regulation of steroidogenesis by visfatin in ovarian follicles in other species, we hypothesized that visfatin also modulates the secretion of steroid and prostaglandin by porcine luteal cells. In the current study, we determined the in vitro effect of visfatin on P_4_, E_2_, PGE_2_, and PGF_2α_ secretion during individual stages of the luteal phase. Moreover, because luteinizing hormone (LH) and INS are known hormones regulating steroid biosynthesis in luteal cells^28,29^, we also evaluated visfatin effect on steroidogenesis in the cells induced by both hormones. The effect of visfatin on steroid synthesis was confirmed by examining the transcript and protein levels of steroidogenic markers (STAR, CYP11A1, HSD3B, and CYP19A1). Additionally, we examined the action of visfatin on the expression of prostaglandin receptors (PTGER2 and PTGFR); INSR; and signalling pathways namely: mitogen-activated protein kinases/extracellular signal-regulated kinase 1/2 (MAPK/ERK1/2), protein kinase B (AKT), and 5′AMP-activated protein kinase (AMPK). Finally, using blocker of enzymatic activity of NAMPT as well as selective pharmacological blockers of INSR, MAPK/ERK1/2, AKT, and AMPK we evaluated the involvement of those signalling pathways in visfatin action on steroid and prostaglandin secretion.

## Results

### The effect of visfatin on basal and LH- and INS-induced P_4_ and E_2_ secretion by porcine luteal cells during the estrous cycle

A two-way analysis of variance (ANOVA) demonstrated that the secretion of P_4_ and E_2_ was affected by the visfatin, FK866, and the interaction of those factors. However, the effect did not occur in all cases. The impact of studied factors depended not only on the studied hormone, the treatment that stimulated steroid hormone secretion, but also on the phase of the estrous cycle. Detailed results of the two-way ANOVA are provided in Supplementary Tables 1 and 2.

**Figure 1:**
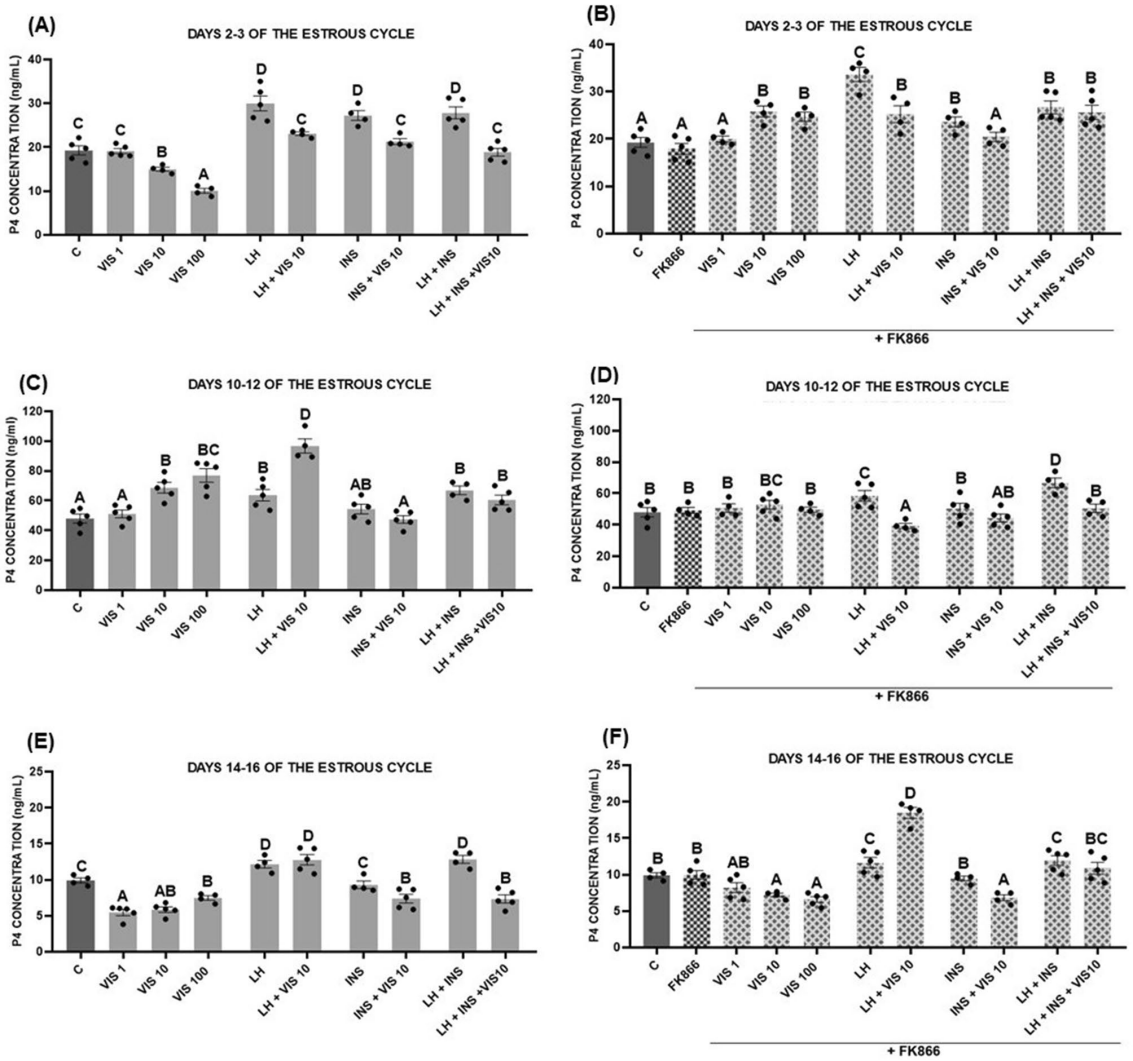
The effect of visfatin (VIS) on basal and luteinizing hormone (LH)- and insulin (INS)-induced progesterone (P_4_) secretion by luteal cells collected from CL on days 2–3 (**A**), 10–12 (**C**), and 14–16 (**E**) of the estrous cycle. Luteal cells were also treated with the blocker FK866 and tested for the effect on P_4_ secretion (**B**, **D**, **F**). The data are presented as the mean ± standard error of the mean (n = 6 replicates). Dots marked individual values indicating the distribution of a range of values. Within each panel, bars/means without a common capital letter differs significantly (*p* < 0.05).

Moreover, post-hoc testing (Tukey's post-hoc test) demonstrated that on days 2–3 of the estrous cycle, visfatin (10 and 100 ng/mL) decreased the P_4_ concentration. We also noted that LH, INS, and LH with INS upregulated the P_4_ level, while the addition of visfatin caused suppression of P_4_ secretion to the control level. Interestingly, we observed no differences between the effects of LH together with INS on P_4_ levels and these factors added separately (*p* < 0.05, Fig. 1A). In the presence of FK866, visfatin (10, 100 ng/mL) enhanced P_4_ release. Interestingly, FK866 did not change the effect of visfatin in combination with LH or INS, but abolished the influence of visfatin added together with LH and INS compared to the effects of these treatments without a blocker (*p* < 0.05, Fig. 1B).

On days 10–12 of the estrous cycle, visfatin (10 and 100 ng/mL) increased P_4_ release. INS alone did not affect P_4_ level, while LH alone and with INS increased P_4_ secretion. Moreover, we observed the additive effect of visfatin and LH: it led to highest the P_4_ level (*p* < 0.05, Fig. 1C). In the presence of FK866, we noticed the inhibitory effect of visfatin added with LH and LH with INS as well as suppression of the action of visfatin added alone at the dose of 10 and 100 ng/mL (*p* < 0.05, Fig. 1D).

In the late luteal phase, on days 14–16 of the estrous cycle, all tested visfatin doses downregulated the P_4_ level. LH added alone and with INS increased P_4_ secretion. Moreover, the addition of visfatin resulted in a decrease in the P_4_ level compared with the action of LH and INS together. Similarly, visfatin administered together with INS lowered the P_4_ level compared with the control and INS, which alone did not affect P_4_ level (*p* < 0.05, Fig. 1E). However, in the presence of FK866, only the effect of visfatin added alone at the concentration of 1 ng/mL was abolished. Also, in the presence of FK866, we noted the stimulation of P_4_ release as a result of the combined administration of visfatin with LH but no effect on the suppression of P_4_ by visfatin in the presence of INS (*p* < 0.05, Fig. 1F).

**Figure 2:**
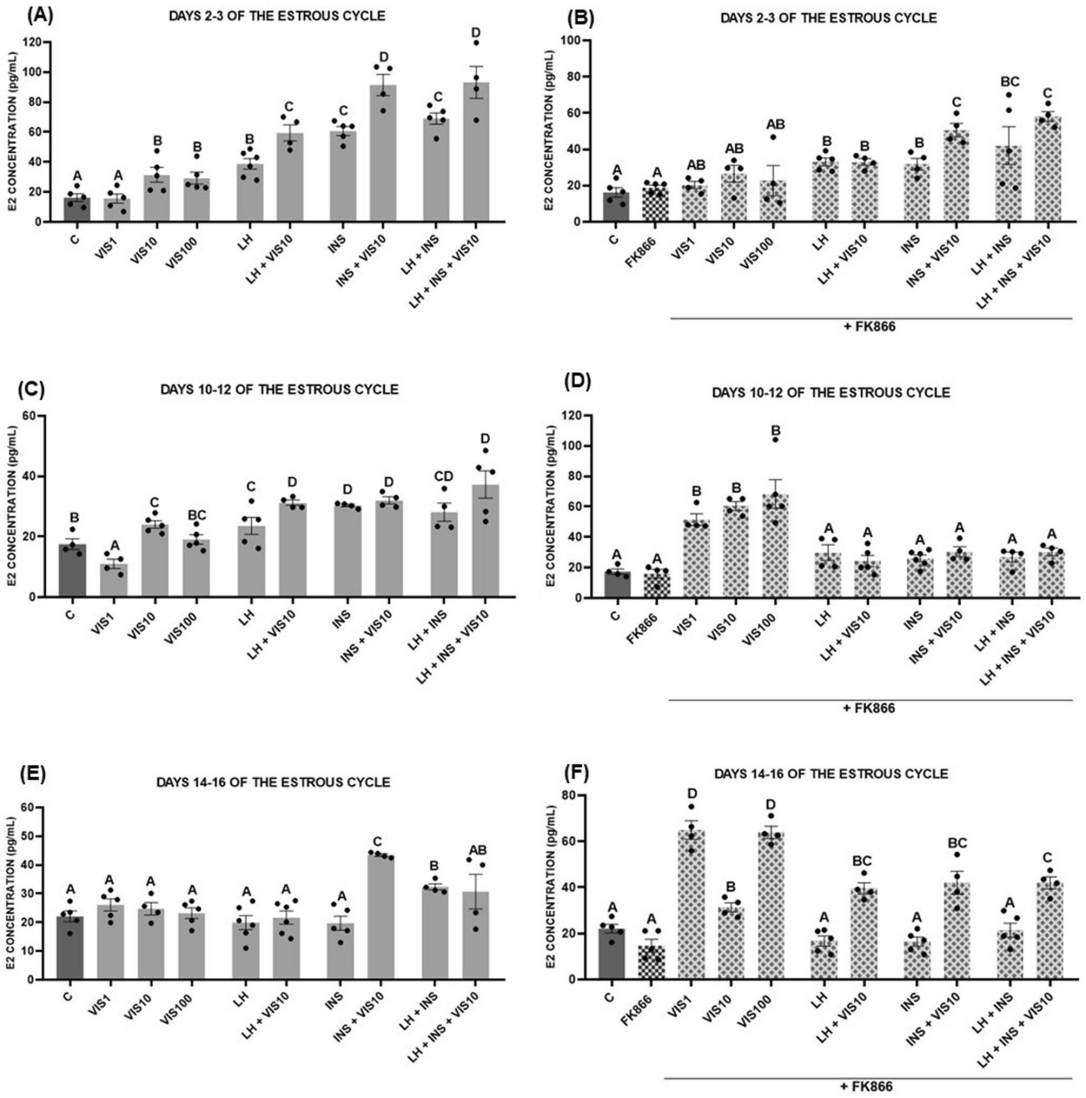
The effect of visfatin (VIS) on basal and luteinizing hormone (LH)- and insulin (INS)-induced estradiol (E_2_) secretion by luteal cells collected from CL on days 2–3 (**A**), 10–12 (**C**), and 14–16 (**E**) of the estrous cycle. Luteal cells were also treated with the blocker FK866 and tested for the effect on E_2_ secretion (**B**, **D**, **F**). The data are presented as the mean ± standard error of the mean (n = 6 replicates). Dots marked individual values indicating the distribution of a range of values. Within each panel, bars/means without a common capital letter differs significantly (*p* < 0.05).

The E_2_ level was higher after the treatment with visfatin (10 and 100 ng/mL) on days 2–3 of the estrous cycle. Similarly, LH and/or INS stimulated E_2_ secretion, and the addition of visfatin with these hormones also increased E_2_ level (*p* < 0.05, Fig. 2A). FK866 abolished the basal visfatin effect on E_2_ secretion at doses of 10 and 100 ng/mL as well as the stimulatory influence of visfatin added together with LH and LH with INS but not with INS alone. The E_2_ levels that were stimulated by combined LH and INS treatment with visfatin were significantly lower in presence of FK866 (*p* < 0.05, Fig. 2B).

On days 10–12 of the estrous cycle, E_2_ secretion was higher after treatment with visfatin at the dose 10 ng/mL, and decreased when visfatin was added at 1 ng/mL. LH and INS stimulated E_2_ release but visfatin enhanced this effect only when administered with LH (*p* < 0.05, Fig. 2C). The use of FK866 alone did not affect E_2_ secretion, but it reversed the inhibitory effect of visfatin at a dose of 1 ng/mL. The visfatin at doses of 1, 10 and 100 ng/mL significantly stimulated E_2_ secretion in the presence of FK866. Moreover, we noted that the effect of visfatin together with LH was also abolished in the presence of FK866 (*p* < 0.05, Fig. 2D).

On days 14–16 of the estrous cycle, visfatin did not affect the E_2_ concentration, except when it was administered together with INS, where it increased E_2_ level compared with the control and INS alone (*p* < 0.05, Fig. 2E). Interestingly, FK866 and visfatin at all tested doses increased E_2_ secretion in this phase of the estrous cycle. Additionally, we observed that in the presence of FK866, visfatin together with LH and LH with INS increased E_2_ levels, which we did not observe without the addition of the blocker. FK866 did not change the effect of visfatin in combination with INS (*p* < 0.05, Fig. 2F).

### The effect of visfatin on the mRNA and protein abundance of steroidogenic markers in porcine luteal cells

**Figure 3:**
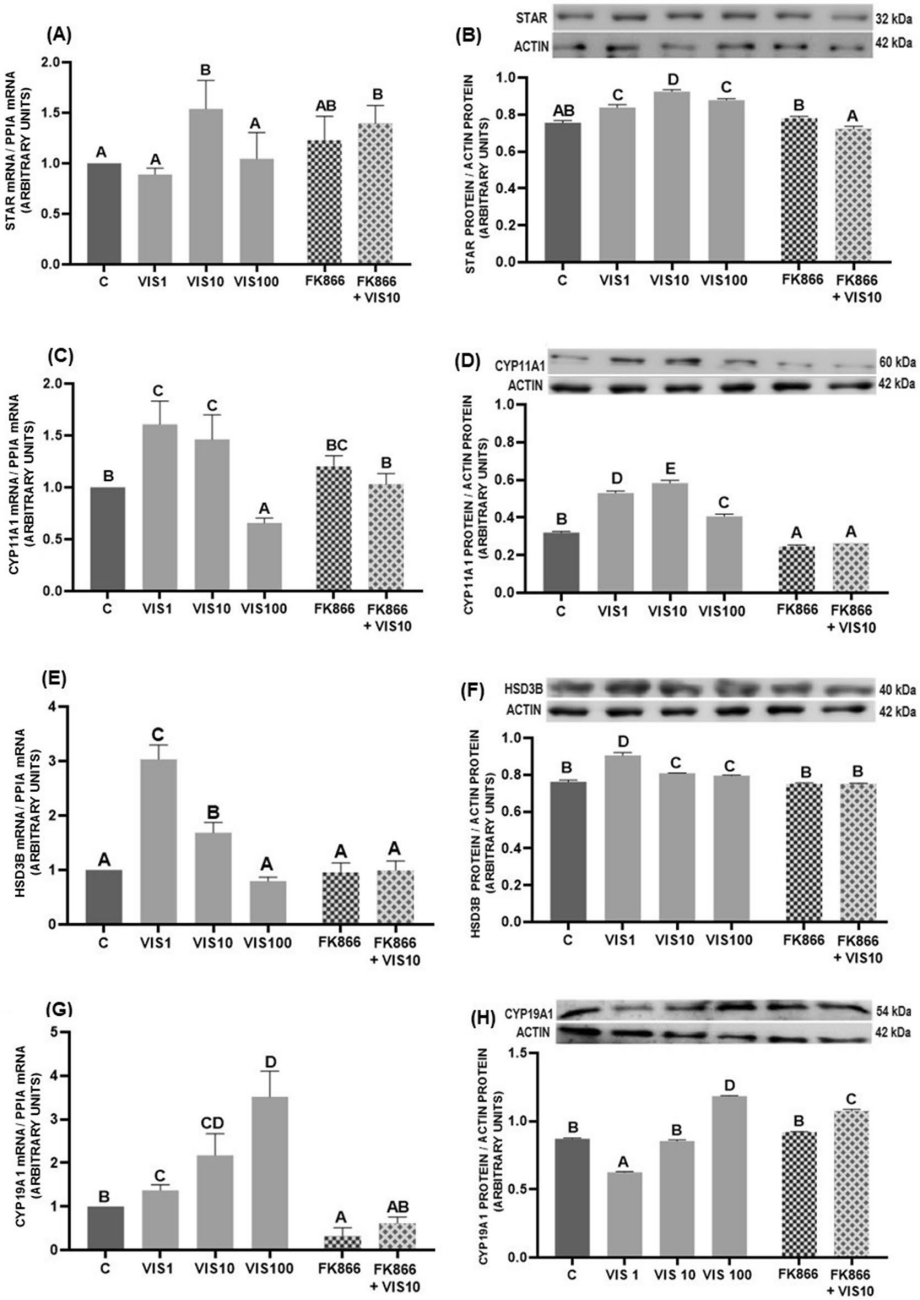
The effect of visfatin (VIS) and its blocker, FK866, on mRNA and protein abundance of steroidogenic markers: steroidogenic acute regulatory protein (STAR) (**A**, **B**), cytochrome P450 family 11 subfamily A member 1 (CYP11A1) (**C**, **D**) hydroxy-delta-5-steroid dehydrogenase (HSD3B) (**E**, **F**), and cytochrome P450 family 19 subfamily A member 1 (CYP19A1) (**G**, **H**) in porcine luteal cells isolated on days 10–12 of the estrous cycle. Abundance of mRNA was expressed as a fold of controls. The protein abundance was analyzed by western blot. The results are shown as representative immunoblots and a bar graph with densitometry measurement of relative target protein content normalized to actin. The data are presented as the mean ± standard error of the mean (n = 6 replicates). Within each panel, bars/means without a common capital letter differs significantly (*p* < 0.05).

We noted that visfatin dependently on the dose used, increased mRNA levels of steroidogenic markers: at the concentration of 10 ng/mL *STAR* (*p* < 0.05, Fig. 3A), at 1 and 10 ng/mL *CYP11A1* and *HSD3B* (*p* < 0.05, Fig. 3C,E), while at all tested doses *CYP19A1* (*p* < 0.05, Fig. 3G). Visfatin decreased *CYP11A1* mRNA level at dose of 100 ng/mL (*p* < 0.05, Fig. 3C). In addition, all tested visfatin doses stimulated the protein abundance of STAR, CYP11A1, and HSD3B (*p* < 0.05, Fig. 3B,D,F). However, CYP19A1 protein expression was decreased after visfatin treatment at the dose of 1 ng/mL but increased at 100 ng/mL (*p* < 0.05, Fig. 3H). Overall, FK866 did not change the expression of studied markers except for CYP11A1 protein (*p* < 0.05, Fig. 3D) and *CYP19A1* mRNA (*p* < 0.05, Fig. 3G), which levels were reduced compared with the control. The addition of the FK866 together with visfatin at the dose of 10 ng/mL resulted in the abolition of the stimulatory effect of visfatin on the mRNA and protein abundance of all examined steroidogenic markers, except for *STAR* mRNA level as well as for CYP19A1 protein levels where treatment with visfatin at this dose and FK866 increased the protein concentrations (*p* < 0.05, Fig. 3A,H).

### The effect of visfatin on prostaglandin secretion by porcine luteal cells during the estrous cycle

**Figure 4:**
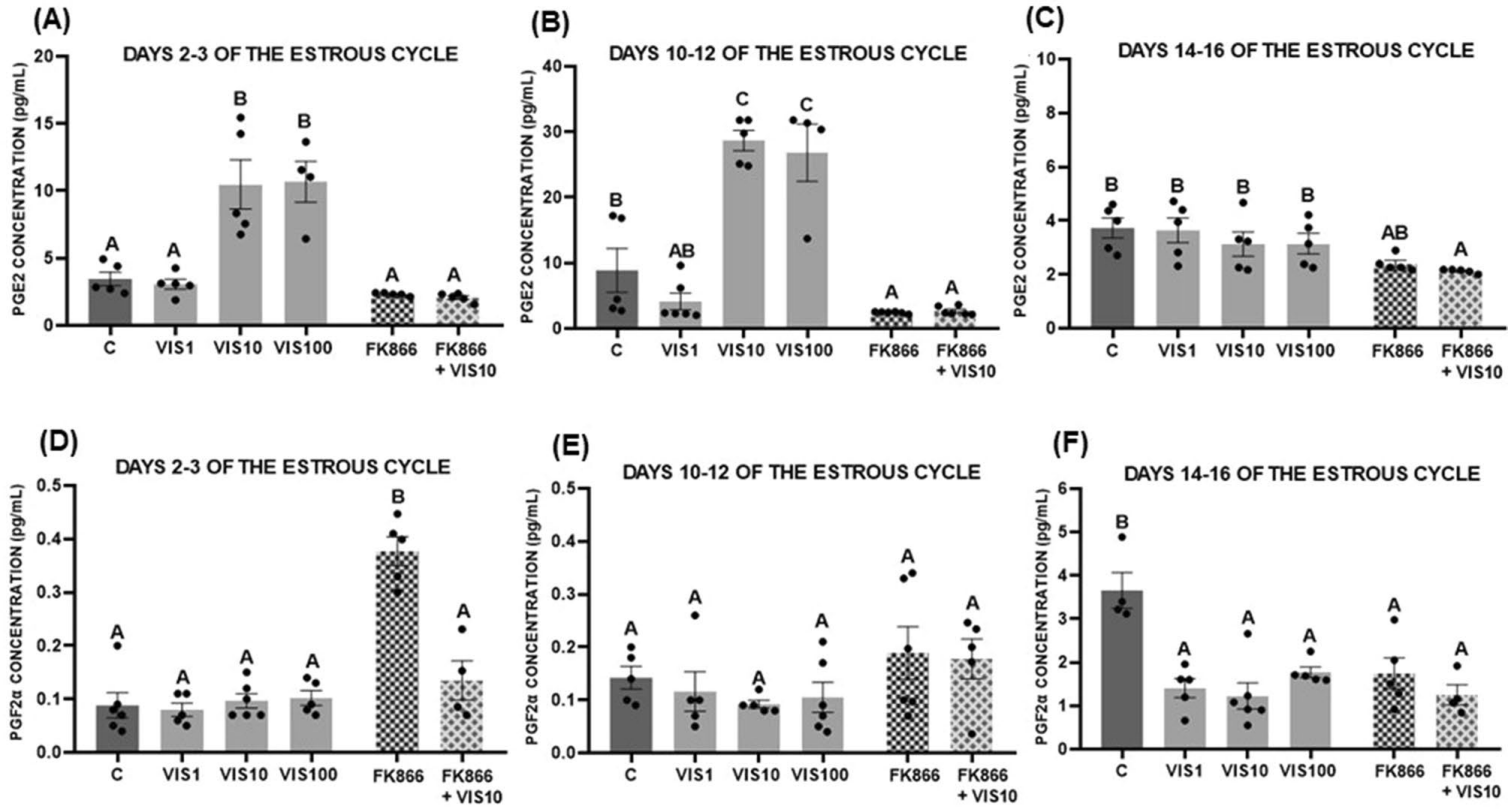
The effect of visfatin (VIS) and its blocker, FK866, on prostaglandin E_2_ (PGE_2_) (**A**–**C**) and prostaglandin F_2α_ (PGF_2α_) (**D**–**F**) secretion by luteal cells collected from CL on days 2–3, 10–12, 14–16 of the estrous cycle. The data are presented as the mean ± standard error of the mean (n = 6 replicates). Dots marked individual values indicating the distribution of a range of values. Within each panel, bars/means without a common capital letter differs significantly (*p* < 0.05).

Secretion of luteotropic PGE_2_ was significantly higher after treatment with visfatin at the doses of 10 and 100 ng/mL on days 2–3 and 10–12 (*p* < 0.05, Fig. 4A,B) of the estrous cycle, while there was no effect on days 14–16 (Fig. 4C). Furthermore, treatment of the cells with visfatin and FK866 abolished the visfatin effect on the PGE_2_ secretion on days 2–3 and 10–12 of the estrous cycle (*p* < 0.05, Fig. 4A,B), whereas on days 14–16 reduced PGE_2_ secretion compared with control and visfatin (10 ng/mL) groups (*p* < 0.05, Fig. 4C).

Regarding PGF_2α_, we noted that visfatin (1–100 ng/mL) decreased its level on days 14–16 of the estrous cycle (*p* < 0.05, Fig. 4F) and had no effect on the other periods studied (Fig. 4D–E). Additionally, FK866 alone increased the PGF_2α_ level on days 2–3 of the estrous cycle (*p* < 0.05, Fig. 4D) and reduced the PGF_2α_ concentration on days 14–16 (*p* < 0.05, Fig. 4F). However, in the former case, the addition of 10 ng/mL visfatin returned PGF_2α_ secretion to the control level (*p* < 0.05, Fig. 4D).

### The effect of visfatin on the expression of prostaglandin receptors in porcine luteal cells

**Figure 5:**
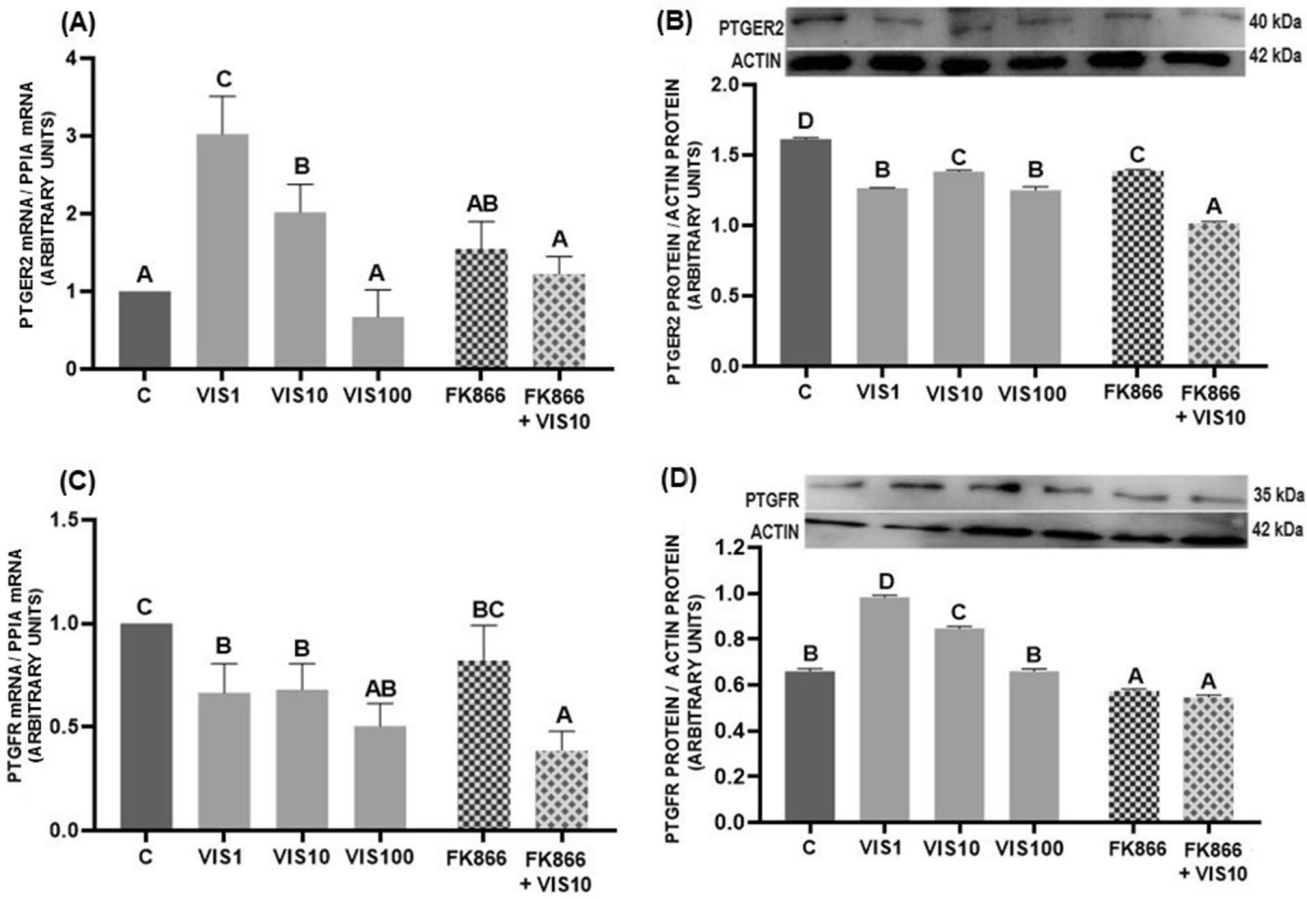
The effect of visfatin (VIS) and its blocker, FK866, on mRNA and protein abundance of the prostaglandin E_2_ receptor (PTGER2) (**A** and **B**) and the prostaglandin F_2α_ receptor (PTGFR) (**C** and **D**) in porcine luteal cells isolated on days 10–12 and 14–16 of the estrous cycle, respectively. Abundance of mRNA was expressed as a fold of controls. The protein abundance was analysed by western blot. The results are shown as representative immunoblots and a bar graph with densitometry measurement of relative target protein content normalized to actin. The data are presented as the mean ± standard error of the mean (n = 6 replicates). Within each panel, bars/means without a common capital letter differs significantly (*p* < 0.05).

Expression of *PTGER2* mRNA was upregulated by visfatin at the dose of 1 and 10 ng/mL (*p* < 0.05, Fig. 5A), while PTGER2 protein abundance was reduced by all visfatin doses (*p* < 0.05, Fig. 5B). FK866 abolished the effect of visfatin on the *PTGER2* mRNA level (*p* < 0.05, Fig. 5A), but reduced PTGER2 protein abundance relative to visfatin (10 ng/mL) and control groups (*p* < 0.05, Fig. 5B).

We observed an opposite effect of visfatin on the PGF_2α_ receptor: at the gene level, visfatin (1–100 ng/mL) decreased PTGFR expression (*p* < 0.05, Fig. 5C), while at the dose of 1 and 10 ng/mL it increased protein abundance (*p* < 0.05, Fig. 5D). FK866 blocked the stimulatory effect of visfatin on PTGFR protein abundance (*p* < 0.05, Fig. 5D), but further decreased *PTGFR* mRNA and PTGER2 protein suppressed by visfatin (*p* < 0.05, Fig. 5B,C).

### The effect of visfatin on activation of INSR, AKT, MAPK/ERK1/2, and AMPK in porcine luteal cells

**Figure 6:**
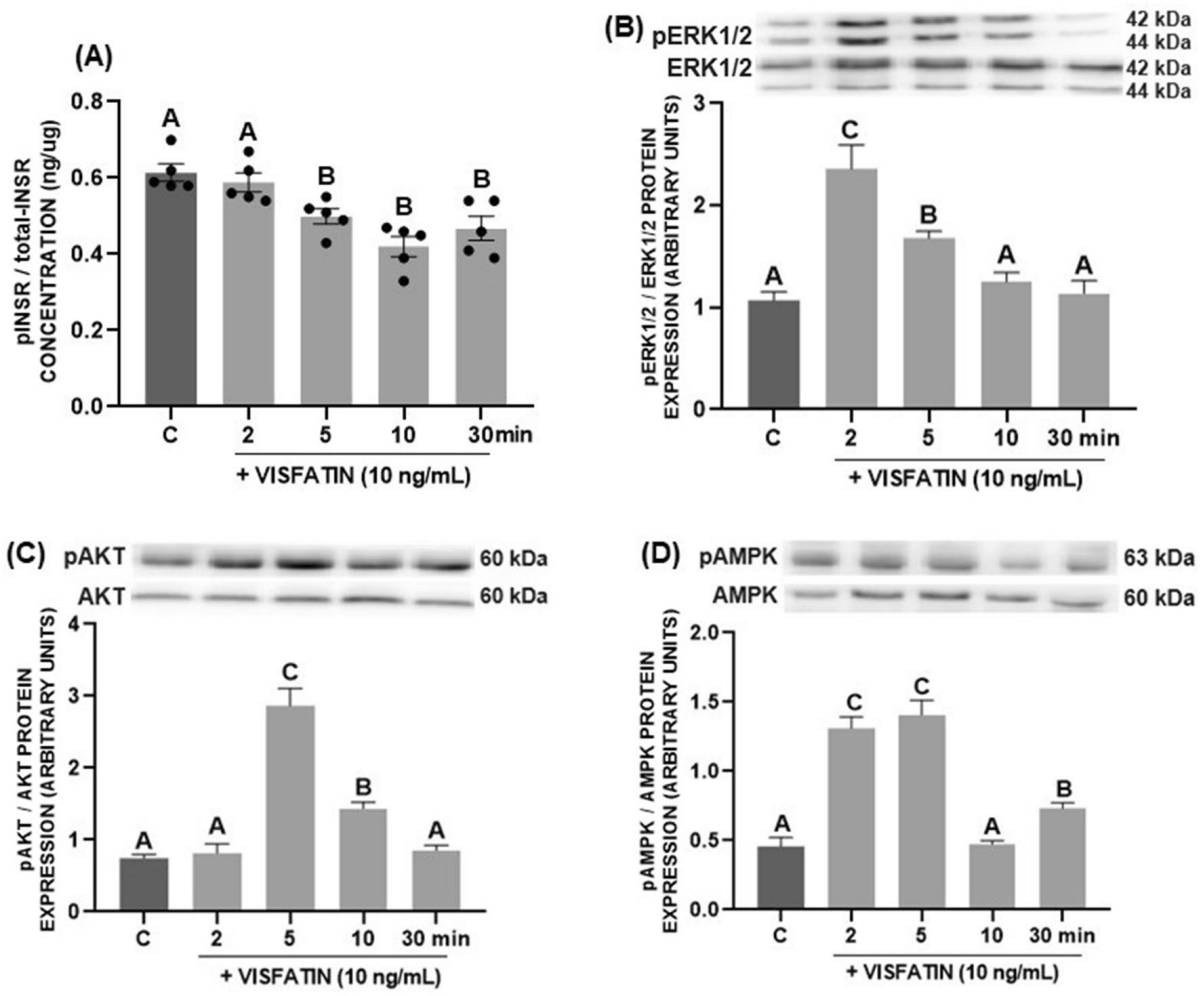
The time-dependent effect of visfatin at the dose of 10 ng/mL on the concentration of phosphorylated and total insulin receptor (INSR) (**A**), and the protein abundance of phosphorylated and total form of extracellular signal-regulated kinase 1/2 (ERK1/2) (**B**), protein kinase B (AKT) (**C**), and 5′AMP-activated protein kinase (AMPK) (**D**) in porcine luteal cells isolated on days 10–12 of the estrous cycle. The concentration of phosphorylated and total INSR in culture medium was measured using enzyme-linked immunosorbent assay. The protein abundance was determined by western blot method. The results are shown as representative immunoblots and a bar graph with densitometry measurement of the phosphorylated form relative to the total form. The data are presented as the mean ± standard error of the mean (n = 6 replicates). Within each panel, bars/means without a common capital letter differs significantly (*p* < 0.05).

We observed that visfatin at 10 ng/mL caused a decrease in the concentration of the phosphorylated form of the INSR after incubation for 5, 10, and 30 min (*p* < 0.05, Fig. 6A). Moreover, we noted activation of MAPK/ERK1/2 after incubation for 2 and 5 min (*p* < 0.05, Fig. 6B); AKT after incubation for 5 and 10 min (*p* < 0.05, Fig. 6C); and AMPK after incubation for 2, 5, and 30 min with visfatin at the dose of 10 ng/mL (*p* < 0.05, Fig. 6D).

### The effect of visfatin on steroid and prostaglandin secretion by porcine luteal cells after treatment with INSR, AKT, MAPK/ERK1/2, and AMPK inhibitors

**Figure 7:**
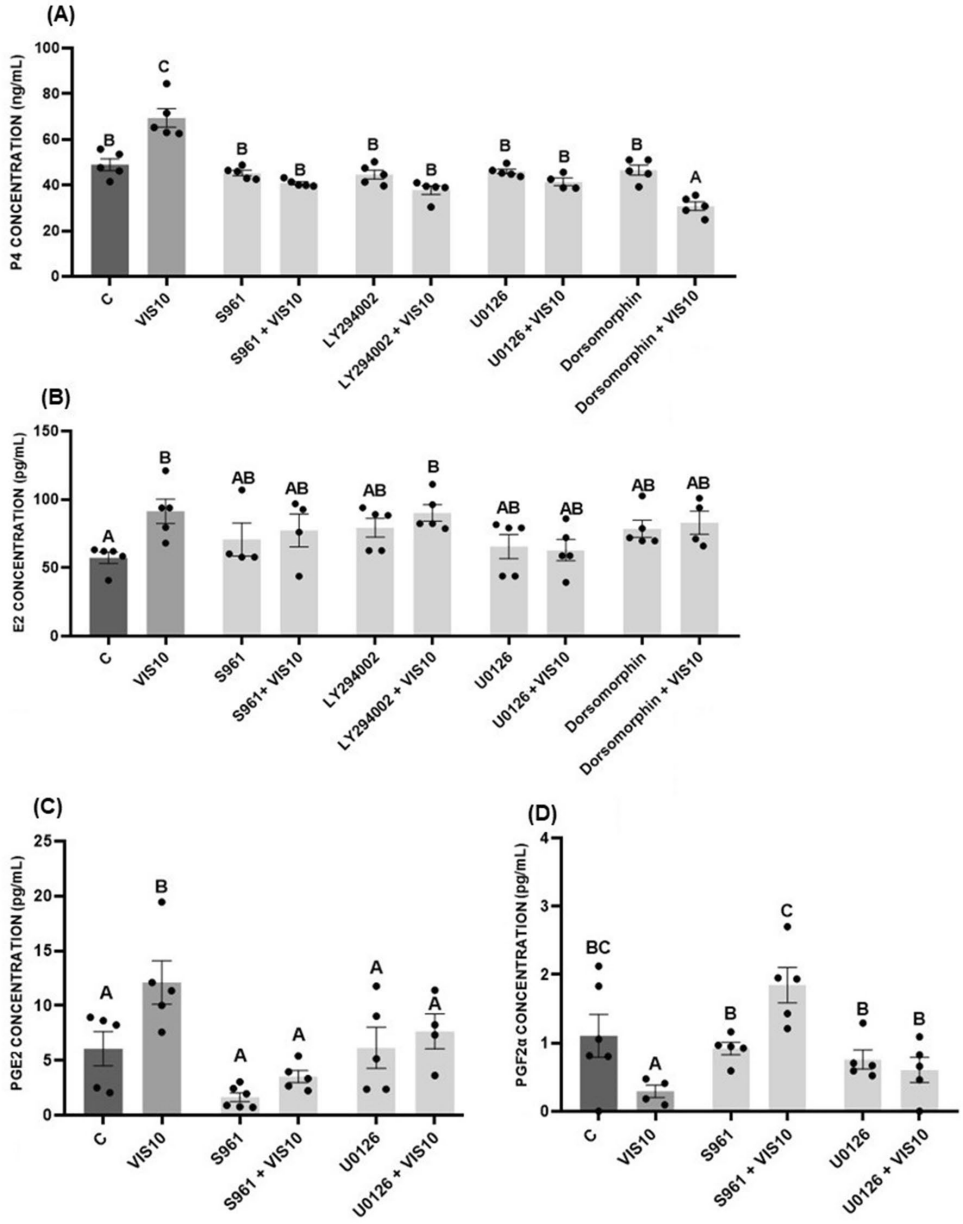
The involvement of the insulin receptor (INSR), mitogen-activated protein kinase/extracellular signal-regulated kinase 1/2 (MAPK/ERK1/2), protein kinase B (AKT), and 5′AMP-activated protein kinase (AMPK) in visfatin (VIS)-mediated regulation of progesterone (P_4_) (**A**), estradiol (E_2_) (**B**), prostaglandin E_2_ (PGE_2_) (**C**), and prostaglandin F_2α_ (PGF_2α_) (**D**) secretion by porcine luteal cells. The cells were isolated on days 10–12 (**A**–**C**) or 14–16 (**D**) of the estrous cycle. To block INSR, AKT, ERK1/2, and AMPK, the pharmacological blockers S961, LY294002, UO126, and Dorsomorphin, respectively, were used. The data are presented as the mean ± standard error of the mean (n = 6 replicates). Dots marked individual values indicating the distribution of a range of values. Within each panel, bars/means without a common capital letter differs significantly (*p* < 0.05).

The stimulatory effect of visfatin on P_4_ secretion was blocked by inhibitors of INSR (S961), AKT (LY294002), MAPK/ERK1/2 (U0126), and AMPK (Dorsomorphin) pathways (*p* < 0.05, Fig. 7A). We noted similar dependence in the case of E_2_ secretion, except for the AKT blocker, which could not counter the effect of visfatin (*p* < 0.05, Fig. 7B). Finally, the use of INSR and MAPK/ERK1/2 inhibitors abolished the effect of visfatin on PGE_2_ and PGF_2α_ secretion (*p* < 0.05, Fig. 7C,D).

## Discussion

The CL is a very dynamic structure; it is formed after ovulation from the Gc and theca cells, which undergo luteinization in just a few days. This is followed by a period of increased P_4_ secretion, aiming to prepare the uterus for embryo implantation. However, in the absence of fertilization, the CL must undergo regression to allow a new cycle to begin. This ensures the proper cyclicity of the ovary^1^. Adipokines are recognized as factors that regulate luteal tissue function in livestock and are identified as mediators linking energy balance and fertility^30^. Consequently, adipokines have been extensively studied within the female reproductive system. One of them, visfatin, has been widely demonstrated to affect steroidogenesis in ovarian follicles in humans^18^, mice^20^, cattle^22,23^, and hens^21^. So far, there has been no attempt to determine its impact on steroid or prostaglandin secretion by porcine luteal cells. We addressed this lacuna and comprehensively demonstrated for the first time that visfatin is an important regulator of hormone secretion in the porcine CL throughout the entire luteal phase, during which hormone secretion undergoes dynamic changes.

Based on our findings, we noted the modulatory (stimulatory or inhibitory) effect of visfatin on luteal steroidogenesis dependent on the phase of estrous cycle. On days 2–3 and 14–16 of the estrous cycle, when P_4_ production is lower^31^, visfatin downregulated P_4_ secretion. Furthermore, after administration with LH and/or INS, visfatin also reduced P_4_ secretion in the early luteal phase, similarly to treatment together with INS and LH with INS in the late luteal phase. Therefore, it seems that visfatin acts as a balancing factor, preventing premature or prolonged excessive P_4_ secretion. In the mid-luteal phase, during the heightened activity of luteal cells^31^, there was an increase in the secretion of this steroid under the influence of visfatin. Moreover, the action of LH with visfatin is additive, leading to high P_4_ production reaching the level of about 100 ng/mL. Our previous studies indicated high expression of visfatin in the middle CL, and we observed that P_4_ itself stimulated visfatin protein expression and secretion by porcine luteal cells in the early and middle luteal phase, suggesting a role for visfatin in CL function^24^. Moreover, we noted stimulation of E_2_ secretion by visfatin alone on days 2–3 and 10–12 of the estrous cycle, and the lack of an effect at the end of the luteal phase. However, visfatin in combination with LH and INS increased the release of E_2_ at the end of the luteal phase as well as with INS on days 2–3 of the estrous cycle. Because E_2_, and more precisely its metabolites, have a beneficial effect on angiogenesis in the CL^2^, which has also been described for other tissues^32^, it may suggest a positive influence of visfatin on the development of the CL.

Our results are in good agreement with the published studies of visfatin’s action on ovarian steroidogenesis. In the CL, the effect of visfatin on steroid secretion has only been determined in water buffalo. That study indicates the stimulatory effect of this adipokine on the P_4_ secretion together with increased mRNA level of *STAR*, *CYP11A1*, and *HSD3B*, which are primarily involved in P_4_ synthesis^23^. In Gc, the visfatin effect depends on the species, the maturity of the animals, and the presence of other stimulators of steroidogenesis, such as insulin growth factor 1 (IGF-1) or follicle-stimulating hormone (FSH). For example, in human primary Gc and the KGN cell line, visfatin increased IGF-1-induced P_4_ and E_2_ secretion, while there were no changes after administration with FSH^18^. Similarly, in water buffalo, visfatin at a dose of 10 ng/mL and also in the presence of IGF-1 stimulated E_2_ secretion and the gene expression of CYP19A1^23^. Subsequently, visfatin increased the release of P_4_ and E_2_, which was associated with an increase in STAR and HSD3B protein in cultured bovine Gc^22^. On the other hand, there are reports on the downregulation of steroidogenesis in hens, and mice. Diot et al.^21^ reported that in hens treatment of Gc with visfatin halved basal and IGF1-induced P_4_ secretion, and this was associated with a reduction in STAR and HSD3B protein abundance. Similarly, Annie et al.^20^ implied that in prepubertal mice, visfatin inhibits ovarian steroidogenesis. Our study also indicated that visfatin influences steroidogenesis in luteal cells in pigs by regulating the expression of enzymes involved in steroid synthesis. Examination of steroidogenic markers expression on days 10–12 of the estrous cycle showed that visfatin increased the level of STAR, CYP11A1, HSD3B, and CYP19A1.

Prostaglandins have an important function in the proper lifespan of the CL. PGE_2_ is a luteotropic factor in many species, including pigs. It maintains P_4_ secretion by luteal cells, and its level is highest until the end of the mid-luteal phase. PGF_2α_, a luteolytic factor, causes apoptosis of luteal cells, which leads to regression of the CL at the end of the luteal phase^4^. We demonstrated that visfatin (10 and 100 ng/mL) increased PGE_2_ secretion almost twofold and threefold on days 2–3 and 10–12 of the estrous cycle, respectively, when the demand for luteal cells is highest. Taking into account these results and the previously mentioned effect on P_4_ and E_2_, visfatin can be classified as a luteotropic agent in porcine luteal tissue. There is limited literature on the regulation of prostaglandin levels by visfatin. Gosset et al.^33^ demonstrated that in human chondrocytes obtained from patients with osteoarthritis, visfatin excessively stimulated PGE_2_ synthesis by increasing the expression of proteins involved in its production. In the context of these studies, the authors concluded that visfatin has a catabolic action in chondrocytes associated with inflammation and it is involved in the pathogenesis of this disease. In the CL, PGE_2_ has more protective mode of action. The effect of visfatin on the PGF_2α_ concentration was noticeable only on days 14–16 of the estrous cycle, when its level was reduced in the presence of all tested visfatin doses. These results could indicate a protective effect of visfatin on luteal cells until the end of the luteal phase in pigs. Interestingly, we observed that FK866 significantly increased the PGF_2α_ secretion on days 2–3 of the estrous cycle; however, the addition of visfatin completely abolished this effect, restoring the PGF_2α_ concentration to the control level. This may suggest that visfatin is essential for maintaining the proper level of this prostaglandin.

Prostaglandins act through specific receptors, for PGF_2α_ it is PTGFR, while PGE_2_ may act through several isoforms of the PTGER. In the porcine CL, PTGER isoform 2 (PTGER2) shows the highest expression^34^. Our results regarding the effect of visfatin on the expression of prostaglandin receptors are complex and differ at the transcript and protein levels. Specifically, visfatin increased *PTGER2* mRNA expression but decreased its protein expression on days 10–12 of the estrous cycle. Similarly, *PTGFR* mRNA expression was reduced after treatment with visfatin, while its protein expression was increased. From a functionality standpoint, protein expression provides more crucial information than gene expression. Nevertheless, our results indicate complex regulation at both the transcriptional and translational levels of these proteins, and one of the reasons for the observed changes may be the concentrations of ligands for these prostaglandin receptors. On days 10–12 of the cycle, the PGE_2_ level was higher after visfatin treatment. This excess amount of PGE_2_ might lead to saturation and desensitization or internalization of the PTGER2 receptor, resulting in a decrease in its protein level^35^. We also noted that visfatin reduced the PGF_2α_ level at the end of the luteal phase; with the decreased ligand concentration, there could be an increase in receptor expression in this phase to maximize PTGFR availability and to enhance PGF_2α_ signalling in the regressing CL. This, in turn, could indicate that despite being a luteotropic factor, as we demonstrated in the first experiment, it also supports luteolysis at the proper time, namely the end of the luteal phase.

Visfatin may affect target cells in two ways: it may bind cell surface receptors, such as earlier mentioned TLR4 and CCR5, and activate intracellular signalling pathways, and it may also act as an enzyme, including as an extracellular ectoenzyme^17^. Visfatin/NAMPT is an enzyme involved in the biosynthesis of NAD+, a crucial cofactor in cellular redox reactions. Its availability can impact cellular functions, including those related to steroidogenesis or prostaglandin signalling^36^. We noted that the presence of FK866 in cultures of luteal cells abolished visfatin’s effects and promoted the opposite effect of visfatin on most of the observed outcomes, such as P_4_ secretion in the early and mid-luteal phases, E_2_ level in the early and late luteal phase, expression of steroidogenic enzymes, and prostaglandin secretion. According to the literature data, NAMPT activity is required for visfatin stimulation of steroid secretion in human Gc^18^. Similarly, in prepubertal mice, NAMPT inhibition by FK866 increased E_2_ secretion and upregulated the expression of CYP11A1, HSD17B, and CYP19A1^20^. However, our results indicate, that the endocrinological function of the porcine CL is not influenced solely by the enzymatic form of visfatin (as NAMPT). In many cases, the action of visfatin added alone or together with LH or INS was not blocked by FK866. These findings indicate another mechanism beyond NAMPT enzymatic activity through which visfatin acts in luteal cells. Hence, we investigated the possible activation of INSR by visfatin and its impact on the phosphorylation of several protein kinases, and whether they could mediate the action of visfatin in porcine luteal cells.

Prior to our study, researchers had found that visfatin activates the MAPK/ERK1/2 pathway in the ovary; in bovine Gc, it visfatin increased phosphorylation of MAPK3/1^22^, while in hen Gc it reduced phosphorylation of MAPK3/1^21^. Reverchon et al.^18^ showed strong activation of ERK1/2, AKT, and p38 upon visfatin treatment in human Gc. In our study, visfatin increased MAPK/ERK1/2, AKT, and AMPK phosphorylation in porcine luteal cells. Furthermore, blocking these kinase signalling pathways revealed their involvement in the stimulatory effect of visfatin on P_4_ secretion on days 10–12 of the estrous cycle. In the case of E_2_ secretion, there was no difference between the influence of LY294002 alone and LY294002 with visfatin on E_2_ level, confirming that AKT is not involved in visfatin-mediated regulation of E_2_ secretion. Considering prostaglandins, we focused on examining the involvement of the MAPK/ERK1/2 pathway, due to the fact that this pathway mainly participates in their synthesis. The fact that suppression of visfatin influenced the secretion of both prostaglandins in response to MAPK/ERK1/2 blockade implies that this pathway is involved in visfatin’s action.

The INS-mimetic properties of visfatin exerted by its direct interaction with the INSR are still a subject of discussion. In human osteoblasts^15^ and mouse pancreatic beta cells^14^, visfatin stimulated INSR as well as insulin receptor substrate 1 and 2 (IRS-1 and IRS-2, respectively). Our recent studies showed stimulation of INSR phosphorylation in porcine anterior pituitary cells; in addition, INSR mediated the effect of visfatin on gonadotropins secretion^37^. In porcine luteal cells, we noted a reduction of the phosphorylated form of INSR, which is contrary to most reports in the literature^14,15,37^. Nevertheless, blocking the INSR signalling pathway abolished the observed effect of visfatin on the secretion of both steroids and prostaglandins, suggesting that reducing INSR phosphorylation is crucial for visfatin to influence the secretory functions of luteal cells. Considering, the relationship between visfatin and INS observed in our study, we suggest that the effect of visfatin may not be related to the activation of the signalling pathway via INSR itself, but rather to its weakening. On days 10–12 of the estrous cycle, the administration of INS suppressed visfatin-stimulated P_4_ secretion. Therefore, blocking INS signalling by decreasing INSR phosphorylation allows visfatin to influence the secretion of steroids. In the human hepatic HEP G2 cell line, Heo et al.^38^ also observed that visfatin reduced the levels of phospho-INSR, phospho-IRS-2, phospho-AKT and phospho-glycogen synthase kinase 3 α/β, which are involved in INS signalling processes.

**Figure 8:**
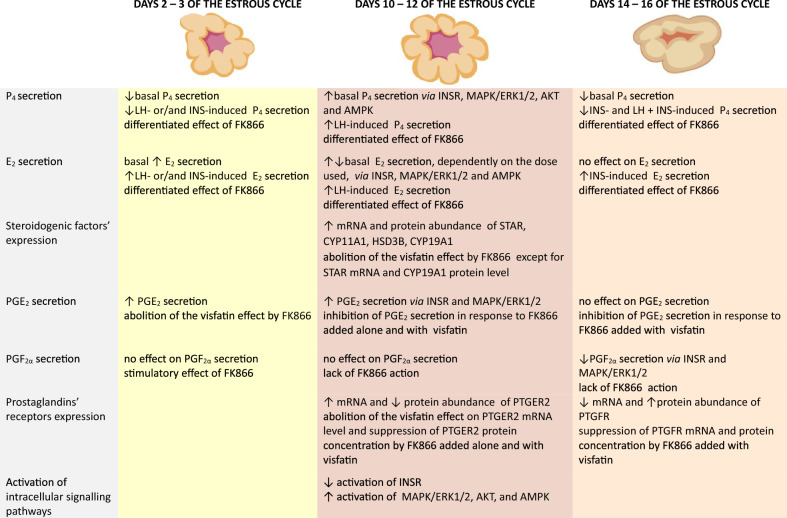
Summary of the visfatin effects on the endocrine function of porcine luteal cells. Abbreviations: FK866—selective blocker of enzymatic activity of visfatin, LH—luteinizing hormone, INS—insulin, P_4_—progesterone, E_2_—estradiol, STAR—steroidogenic acute regulatory protein, CYP11A1—cytochrome P450 family 11 subfamily A member 1, HSD3B—hydroxy-delta-5-steroid dehydrogenase, CYP19A1—cytochrome P450 family 19 subfamily A member 1, PGE_2_—prostaglandin E_2_, PGF_2α_—prostaglandin F_2α_, PTGER2—receptor of PGE_2_, PTGFR—receptor of PGF_2α_, INSR—insulin receptor, MAPK/ERK1/2—mitogen-activated protein kinases/extracellular signal-regulated kinase 1/2, AKT—protein kinase B, AMPK—5′AMP-activated protein kinase.

In summary, we showed that visfatin exerts an effect on the endocrine function of porcine luteal cells by influencing the secretion of steroids and prostaglandins and the expression of steroidogenic markers and prostaglandin receptors (Fig. 8). The action of visfatin depends on the phase of the estrous cycle and is modulated by the presence of LH and INS. It exerts its effect not only through the enzymatic activity of NAMPT, but also through the INSR and MAPK/ERK1/2, AKT, and AMPK. Our results indicate that visfatin may be one of important factors regulating of CL physiology in livestock.

## Material and methods

### Animals and sample collection

The research was carried out on mature cross-breed gilts (Large White × Polish Landrace) at the age of 7–8 months and weighing 140–150 kg from local slaughterhouse in Poland. Samples were collected from pigs intended for commercial purposes and meat processing. The animals were used in accordance with the Act of the 15th of January 2015 (Journal of Laws Dz. U. 2015 No. item 266) on the Protection of Animals Used for Scientific or Education Purposes and Directive 2010/63/EU of the European Parliament and the Council of the 22nd of September 2010 on the Protection of Animals used for Scientific Purposes. Based on this directive, the studies did not required the approval of the relevant ethics committee for experiments on animals.

To determine the effect of visfatin on the endocrine function of porcine CL, we conducted a series of in vitro cultures of luteal cells (six independent in vitro cultures of luteal cells, biological replications, n = 6 per group) isolated from CL on days 2–3 (early luteal phase, formation of the CL), days 10–12 (mid-luteal phase, the highest activity of the CL and production of P_4_), and days 14–16 (late luteal phase, regression of the CL) of the estrous cycle. For each in vitro culture of luteal cells, a subsequent biological replication of the experiment utilized CLs from 5 to 7 pigs. The phases of the estrous cycle were confirmed based on the characteristics of ovarian morphology^39^. Within a few minutes after slaughter, the ovaries were removed, placed in phosphate-buffered saline (PBS, pH 7.4, 4 °C) with a mixture of antibiotics, and transported on ice to the laboratory within 1–1.5 h.

### In vitro luteal cell cultures

Luteal cell isolation and the in vitro cell culture were performed by using the same methodology as Rytelewska et al.^40^. In brief, CLs were dissected, minced mechanically, and then enzymatically digested with 0.1% collagenase type V in Hank’s Balanced Salt Solution (pH 7.4). We separated small and large luteal cells from CLs and non-steroidogenic cells, such as endothelial cells. Erythrocytes have been removed during isolation using a special buffer for the red blood cell lysis. The cells were counted after isolation, and their viability was determined with a trypan blue exclusion test. The mean viability of the cells was 95.60% ± 1.54%. Only luteal cells were counted to inoculate the appropriate amount needed for the experiments. The cells were cultured in 6-well culture plates at the final concentration of 2 × 10^6^ cells/well (2 mL of medium per well) for western blot analysis, in 24-well plates at a concentration of 2.5 × 10^5^ cells/well (1 mL of medium per well) for radioimmunoassay (RIA) and enzyme-linked immunosorbent assay (ELISA), and in 96-well plates at a concentration of 9 × 10^4^ cells/well (200 µL of medium per well) for transcript levels measured by real-time PCR in a humidified incubator (terms: 37 °C, 95% air, 5% CO_2_; Binder CB160, DanLab, Poland). Cells were suspended in Ham’s F-12 medium enriched with 0.268% sodium bicarbonate, 10% fetal bovine serum, 1% bovine serum albumin (BSA), and a mixture of antibiotics. Next, the cells were precultured for 48 h, and then switched to a fresh medium containing 1% FBS and treated with appropriate hormones/markers. The current study comprised six experiments (Supplementary Table 3).

*Experiment 1*: In the first experiment, we aimed to determine the basal and LH- and INS-induced effect of visfatin on P_4_ and E_2_ secretion by the porcine CL during the luteal phase (days 2–3, 10–12, 14–16 of the estrous cycle). Cells were treated with visfatin (cat. no. 8424-VF-050, Bio-Techne, Minneapolis, MN, USA) at the dose of 1, 10 and 100 ng/mL, which we chose based on our previous study^37,41^. Moreover, cells were treated with visfatin (10 ng/mL) together with LH (100 ng/mL, from human pituitary, cat. no. L6420, Sigma-Aldrich, St. Louis, MO, USA) or INS (10 ng/mL, from porcine pancreas, cat. no. I5523, Sigma-Aldrich, St. Louis, MO, USA) or with LH (100 ng/mL) and INS (10 ng/mL). Doses of LH we chosen based on a previous paper Kurowska et al.^7^, while the INS concentration was based on the study by Gavin et al.^42^. Additionally, we used FK866 (10 nM, cat. no. F8557, Sigma-Aldrich, St. Louis, MO, USA) with all mentioned hormones; the dose of FK866 we established based on the study by Reverchon et al.^18^. Importantly, FK866 was added already during the seeding of luteal cells, and changing the medium to fresh with the tested hormones. After incubation for 24 h, the culture medium was collected and stored in − 20 °C for further measurement of P_4_ with RIA and E_2_ with ELISA.

*Experiment 2*: We chose one phase of the estrous cycle (days 10–12) to evaluate whether visfatin regulates steroid synthesis by influencing the expression of markers involved in this process in the CL. Due to the fact that the production of steroids, especially P_4_, is the highest in mid-luteal phase, we decided to check the expression of steroidogenic factors on days 10–12 of the estrous cycle. Luteal cells were treated with visfatin (1–100 ng/mL) and the FK866 blocker alone or with visfatin at the dose of 10 ng/mL. After incubation for 24 h, cell lysates were collected to examine STAR, CYP11A1, HSD3B, and CYP19A1 mRNA and protein expression by real-time PCR and western blot, respectively.

*Experiment 3*: In this experiment, we examined the effect of visfatin on the secretion of prostaglandins by luteal cells during the entire luteal phase (days 2–3, 10–12, and 14–16 of the estrous cycle). Cells were treated with visfatin (1–100 ng/mL) and FK866 alone or with visfatin at the dose of 10 ng/mL. Following incubation, medium was collected to measure the PGE_2_ and PGF_2α_ levels with commercially available ELISA kits.

*Experiment 4*: Based on the results obtained from Experiment 3, we selected one phase of the estrous cycle where visfatin exerts an effect on prostaglandins to check the expression of prostaglandin receptors. Thus, we examined PTGER2 expression on days 10–12 and PTGFR expression on days 14–16 of the estrous cycle. Luteal cells were stimulated with visfatin (1–100 ng/mL) as well as the FK866 alone or with visfatin at the dose of 10 ng/mL. After incubation for 24 h, cell lysates were collected to examine PTGER2 and PTGFR mRNA and protein expression with real-time PCR and western blot, respectively.

*Experiment 5*: We determined the visfatin effect on the activation of INSR and several intracellular signalling pathways in the CL on days 10–12 of the estrous cycle. For this purpose, luteal cells were incubated with visfatin at the dose of 10 ng/mL for 2, 5, 15, and 30 min. Then, medium was collected to examine the phosphorylated and total forms of INSR by ELISA, and cell lysates were collected to examine protein expression of the phosphorylated and total forms of MAPK/ERK1/2, AKT, and AMPK using western blot.

*Experiment 6*: In the final experiment, we evaluated the involvement of INSR and MAPK/ERK1/2, AKT, and AMPK in visfatin’s action on steroid and prostaglandin secretion. We selected only one stage of the estrous cycle based on results obtained in *Experiments 1* and *3*; P_4_, E_2,_ and PGE_2_ secretion was determined on days 10–12, and PGF_2α_ secretion was determined on days 14–16 of the estrous cycle. Cells were treated with the pharmacological blockers of INSR (S961, 1 µM), AKT (LY294002, 20 µM), MAPK/ERK1/2 (U0126, 10 µM), or AMPK (Dorsomorphin, 10 µM). We chose the doses of these blockers based on the studies by Elliot et al.^43^, Zhao et al.^44^, and Reverchon et al.^18^ for S961, LY294002, U0126, and Dorsomorphin, respectively. Luteal cells were preincubated with blockers for 1 h and then we added visfatin at the concentration of 10 ng/mL. After incubation for 24 h, culture medium was collected to measure P_4_ secretion with RIA, and E_2_, PGE_2_, and PGF_2α_ secretion with ELISA.

### RIA

The concentrations of P_4_ in the culture media were determined by RIA with tritium labelling (^3^H, a source of β-radiation) according to the method described by Ciereszko et al.^45^. The specificity of the antibodies against P_4_ (SO/91/4) was previously reported^45^. Before the main analysis, a preliminary test was performed to determine the optimal dilutions of the anti-P_4_ antibody and culture medium to ensure the most effective detection range for the assay. As a result, an antibody dilution of 1:3000 and a culture medium sample dilution of 1:500 were used. The probe radioactivity levels were measured using a Hidex 300 SL scintillation counter (Hidex Oy, Turku, Finland) with the Microwin 2000 software (Mikrotek Laborsysteme GmbH, Overath, Germany). All standards were run in triplicate and all culture medium samples were run in duplicate. The mean antibody binding was 27.55% ± 2.38%. The sensitivity of the assay was 1 pg/mL, while the range of the standard curve was 1–1000 pg/mL. The P_4_ concentration in each sample was determined from the standard curves, which were plotted as polynomial trend lines representing the count per minute (CPM) values standardized against a blank probe for each standard solution versus their respective concentrations. The intra- and inter-assay coefficients of variation were 3.98% ± 0.99% and 9.63% ± 2.02%, respectively.

### ELISA

The concentrations of E_2_, PGE_2,_ and PGF_2α_ as well as phospho-INSR and total-INSR in culture media were determined by using commercially available ELISA kits according to the manufacturers’ protocols. Supplementary Table 4 provides more details about the assays used in this study. The samples were run in duplicate within the same assay. Absorbance values were measured at 450 nm using a Varioskan LU Multimode Microplate Reader and the SkanIt Software 6.1.1 (Thermo Fisher Scientific, MA, USA).

### Real-time PCR

TaqMan gene expression assays (Applied Biosystems, Carlsbad, CA, USA) were employed to quantify the mRNA expression of markers implicated in steroid synthesis, as well as prostaglandin receptors. Total RNA isolation and cDNA synthesis were performed according to the TaqMan Gene Expression Cells-to-CT Kit protocol (cat. no. AM1728, Applied Biosystems, Carlsbad, CA, USA). The RNA and cDNA concentrations were determined based on optical density at 260 and 280 nm. Amplifications were executed using the StepOnePlus system (Applied Biosystems, Carlsbad, CA, USA) under the manufacturer’s instructions, utilizing TaqMan-specific primers for *STAR* (assay ID: Ss03381250_u1), *CYP11A1* (assay ID: Ss03384849_u1), *HSD3B* (assay ID: Ss03391752_m1), *CYP19A1* (assay ID: Ss03384876_u1), *PTGER2* (assay ID: Ss03374177_g1), and *PTGFR* (assay ID: Ss03393819_s1). The final 20-µL reaction volume comprised the TaqMan Gene Expression Master Mix and 50 ng of cDNA. Relative gene expression was normalized against the reference gene *PPIA*^7,46^ (assay ID: Ss03394782_g1). The relative gene expression levels were determined by following the method outlined by Livak and Schmittgen^47^, using the comparative cycle threshold (2^-ΔΔCt^) approach.

### Western blot

Luteal cells were lysed using Tissue Protein Extraction Reagent (cat. no. 78510, Thermo Fisher Scientific, MA, USA) with addition of protease and phosphatase inhibitors. Equal quantities of lysates (30 μg protein/sample) with Laemmli buffer (cat. no. 23225, Sigma-Aldrich, MO, USA) were denatured at 95 °C for 5 min, then separated in 10% sodium dodecyl sulphate–polyacrylamide gels, and transferred onto polyvinylidene fluoride membranes (cat. no. IPVH00010, Sigma-Aldrich, MO, USA). The membranes were incubated in 0.02 M Tris-buffered saline with Tween 20 (TBST) containing 5% BSA for 1 h at 20–22 °C to block nonspecific protein binding. Subsequently, the membranes were incubated overnight at 4 °C with primary antibody. Supplementary Table 5 provides detailed information about antibodies used for this method. The next day, following TBST washes, the membranes were incubated with horseradish peroxidase-conjugated antibody (diluted at 1:1000) (Supplementary Table 5) for 1 h at 20–22 °C. Chemiluminescence detection, using the Immobilon Western Chemiluminescent HRP Substrate (cat. no. WBKLS0500, Sigma-Aldrich, MO, USA), revealed signals that were visualized using the ChemiDoc™ imagining system (Bio-Rad, CA, USA). Actin served as a loading control. The ImageJ software (US National Institutes of Health, Bethesda, MD, USA) was used for densitometric analysis of the protein bands. Representative blots are included as Supplementary Fig. 1.

### Statistical analysis

Statistical analysis was performed using GraphPad Prism 8.0.1 (GraphPad Software, Inc., San Diego, CA, USA). All experimental data are presented as mean ± standard error of the mean of experiments which were performed in six replicates (n = 6). Extreme values were rejected according to the three-sigma rule^48^. The data were evaluated to determine whether they met the assumptions of normality (Shapiro–Wilk test) and homogeneity of variances (Levene’s test) and then analysed with one-way ANOVA followed by Tukey’s test. Statistically significant differences (*p* < 0.05) are indicated by different letters (Supplementary Table 6). Additionally, we performed two-way ANOVA to examine differences in steroid secretion levels with two independent variables (main factors): VIS and FK866, and the interaction of those factors, i.e. VIS*FK866 for each experimental setup and phase of the estrous cycle (Supplementary Tables 1 and 2).

### Ethics declarations and approvals for animal experiments

Ovaries were a by-product from animals intended for research or commercial purposes (meat processing). This study did not require the approval of the ethics committee for experiments on animals, because the slaughter of animals, the collection of biological material, and the transport of material to the laboratory were carried out in accordance with the Polish Act on the Protection of Animals Used for Scientific or Educational Purposes of January 15, 2015 (Journal of Laws Dz.U. 2015 No. item 266) and the European Communities Council Directive 2010/63/UE of September 22, 2010, on the protection of animals used for scientific purposes.

### Supplementary Information

Supplementary Figures.

Supplementary Table 1.

Supplementary Table 2.

Supplementary Table 3.

Supplementary Table 4.

Supplementary Table 5.

Supplementary Table 6.

## Data Availability

Data is provided within the manuscript or supplementary information files.
