# Supplementary material for: In vitro effect of visfatin on endocrine functions of the porcine corpus luteum
Source: Sci Rep. 2024 Jun 26;14:14780. doi: 10.1038/s41598-024-65102-4 (PMC11208563; doi:10.1038/s41598-024-65102-4)
Supplement: Supplementary file 1 — Supplementary Figures. [file 41598_2024_65102_MOESM1_ESM.pdf]

In vitro effect of visfatin on endocrine functions of the porcine corpus luteum

Ewa Mlyczyńska, Edyta Rytelewska, Ewa Zaobidna, Natalia Respekta, Grzegorz Kopij, Kamil Dobrzyń, Marta Kieżun, Nina Smolińska, Tadeusz Kamiński, Agnieszka Rak

Representative original blots for the expression of steroidogenic enzymes under the influence of visfatin included in **Figure 3B**.

STAR

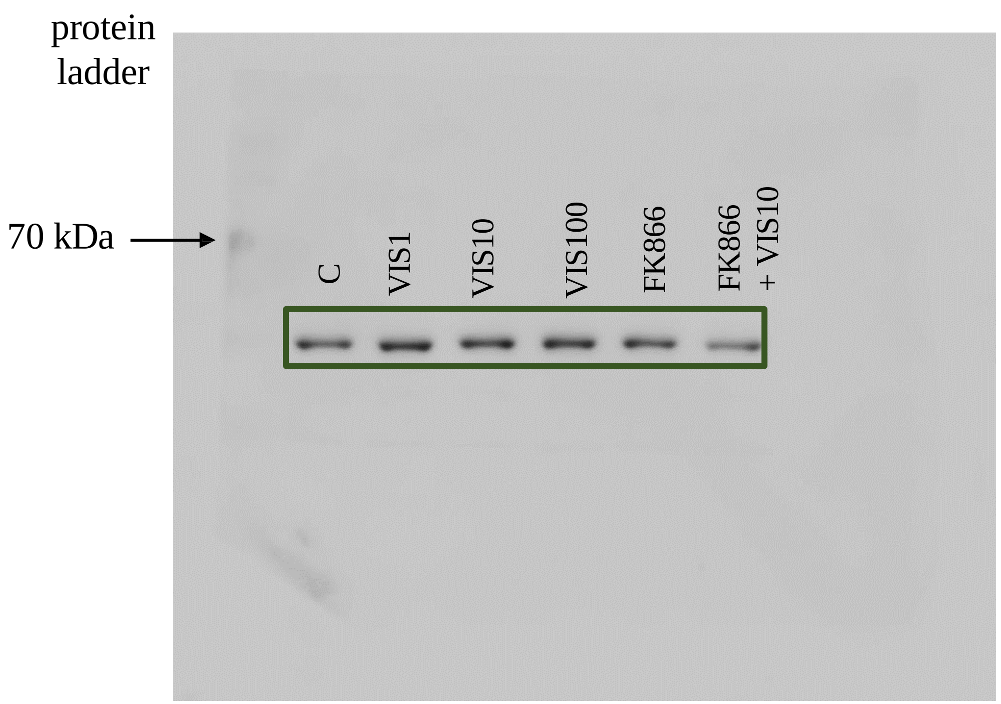

ACTIN

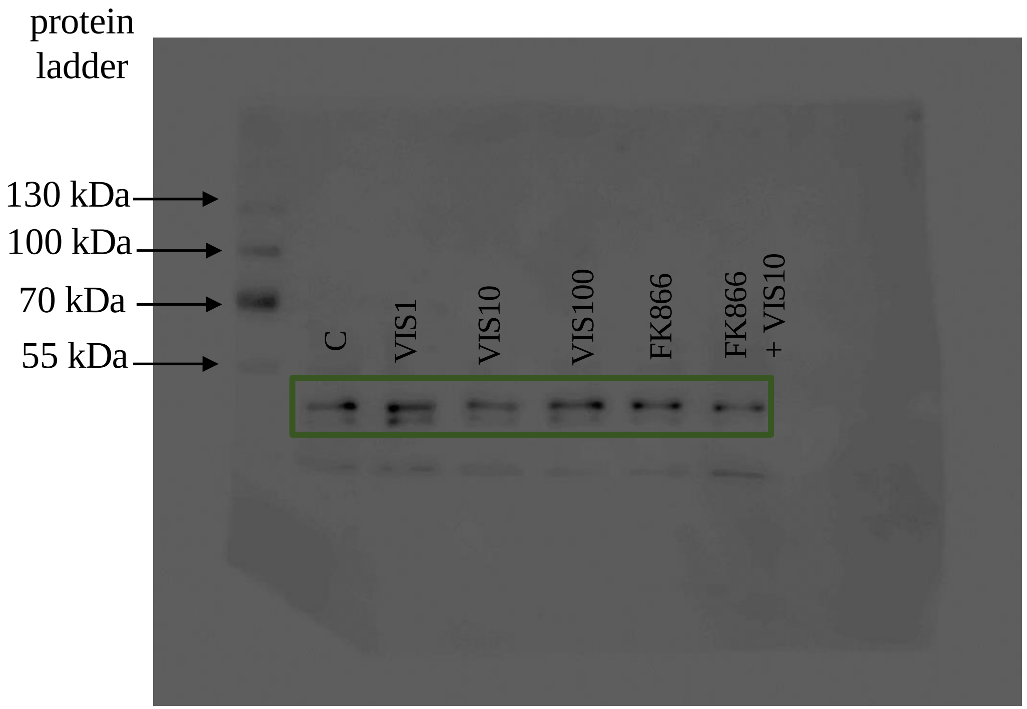

In vitro effect of visfatin on endocrine functions of the porcine corpus luteum

Ewa Mlyczyńska, Edyta Rytelewska, Ewa Zaobidna, Natalia Respekta, Grzegorz Kopij, Kamil Dobrzyń, Marta Kieżun, Nina Smolińska, Tadeusz Kamiński, Agnieszka Rak

Representative original blots for the expression of steroidogenic enzymes under the influence of visfatin included in **Figure 3D**.

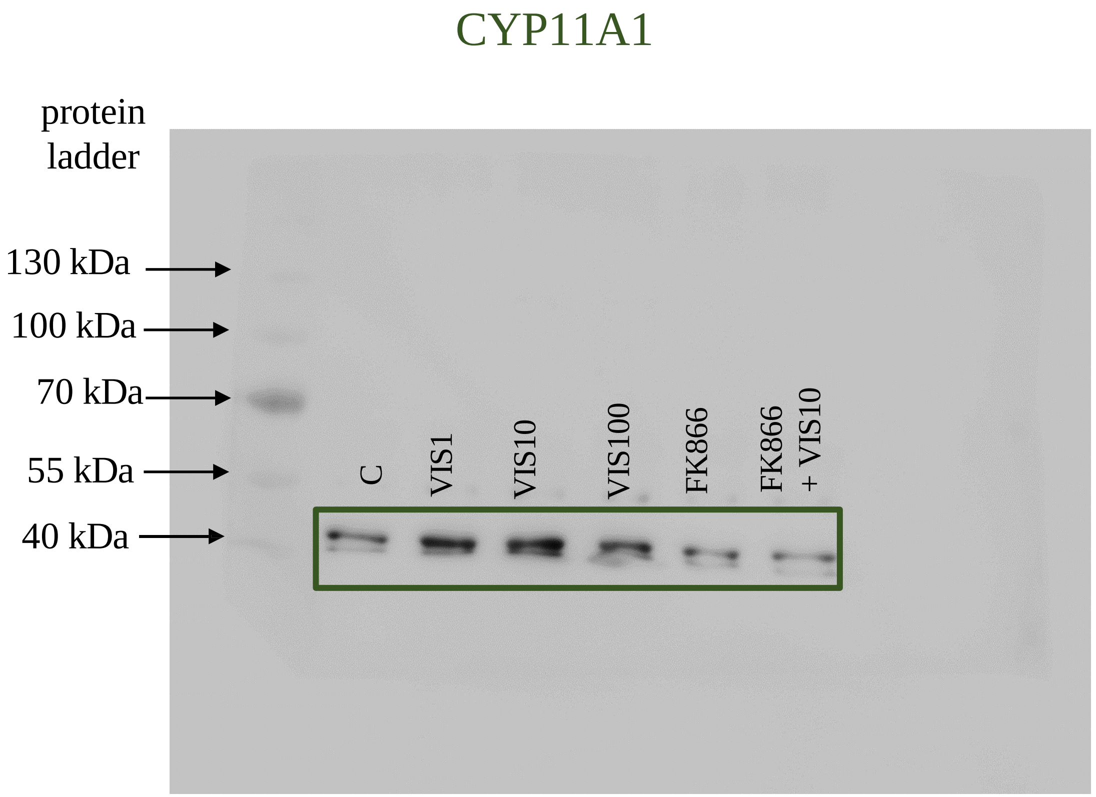

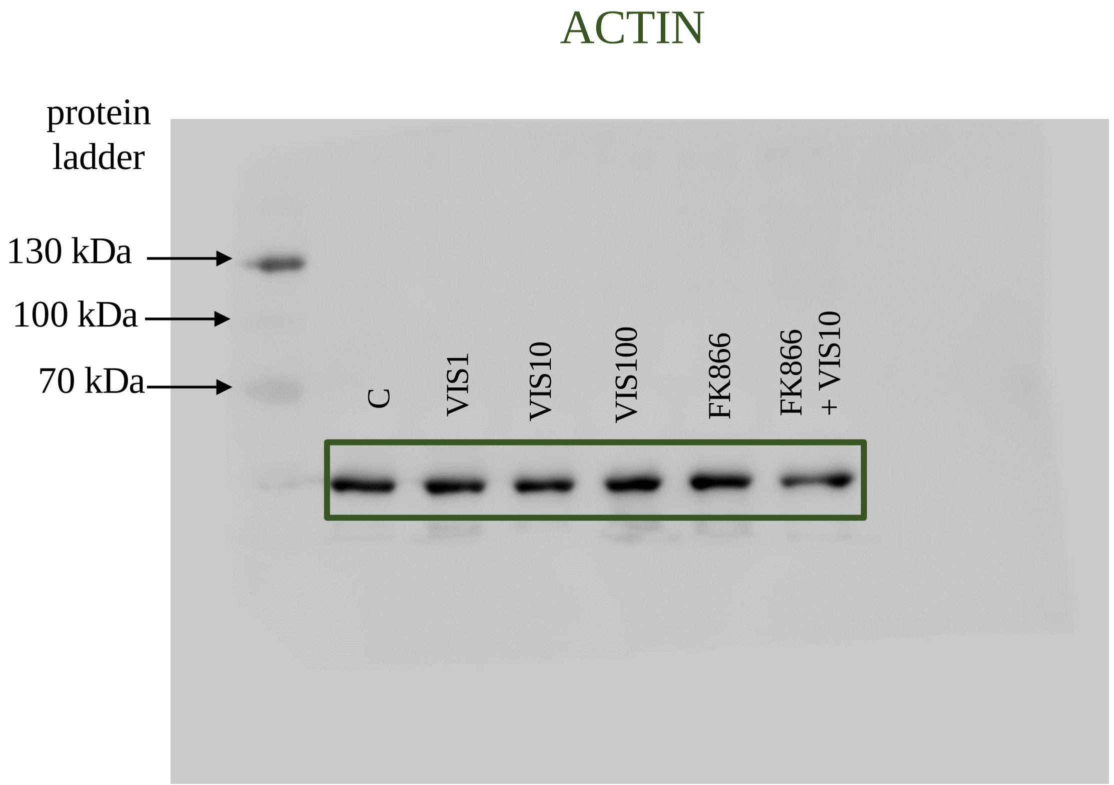

In vitro effect of visfatin on endocrine functions of the porcine corpus luteum

Ewa Mlyczyńska, Edyta Rytelewska, Ewa Zaobidna, Natalia Respekta, Grzegorz Kopij, Kamil Dobrzyń, Marta Kieżun, Nina Smolińska, Tadeusz Kamiński, Agnieszka Rak

Representative original blots for the expression of steroidogenic enzymes under the influence of visfatin included in **Figure 3F**.

HSD3B

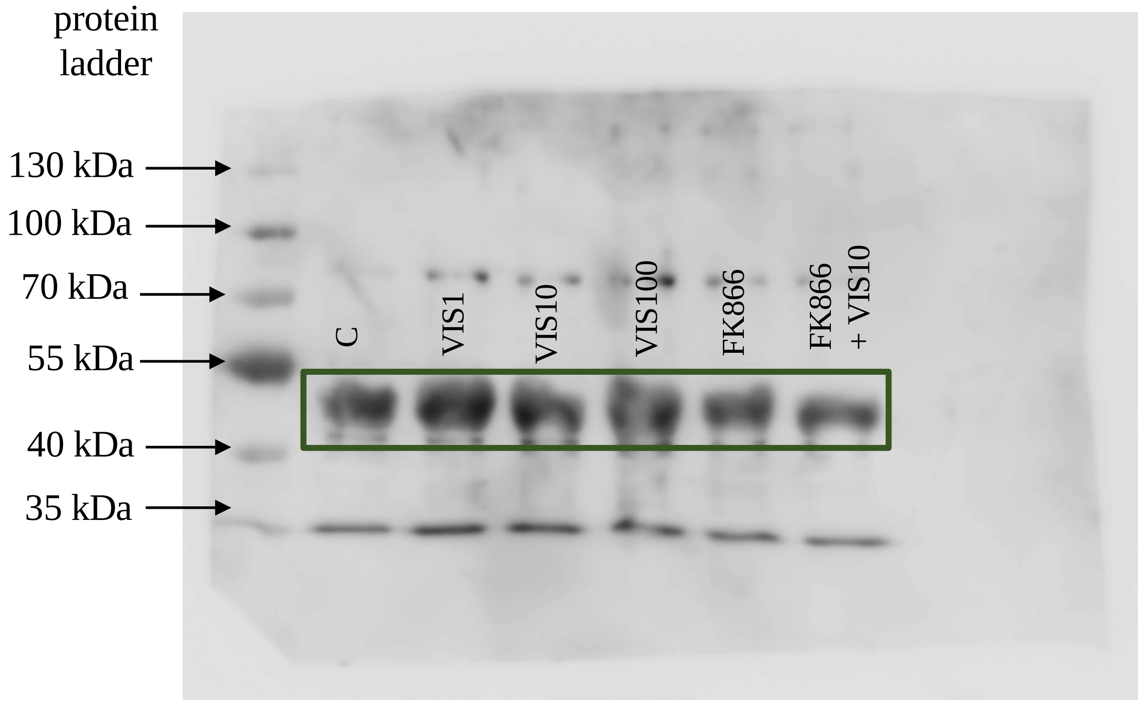

ACTIN

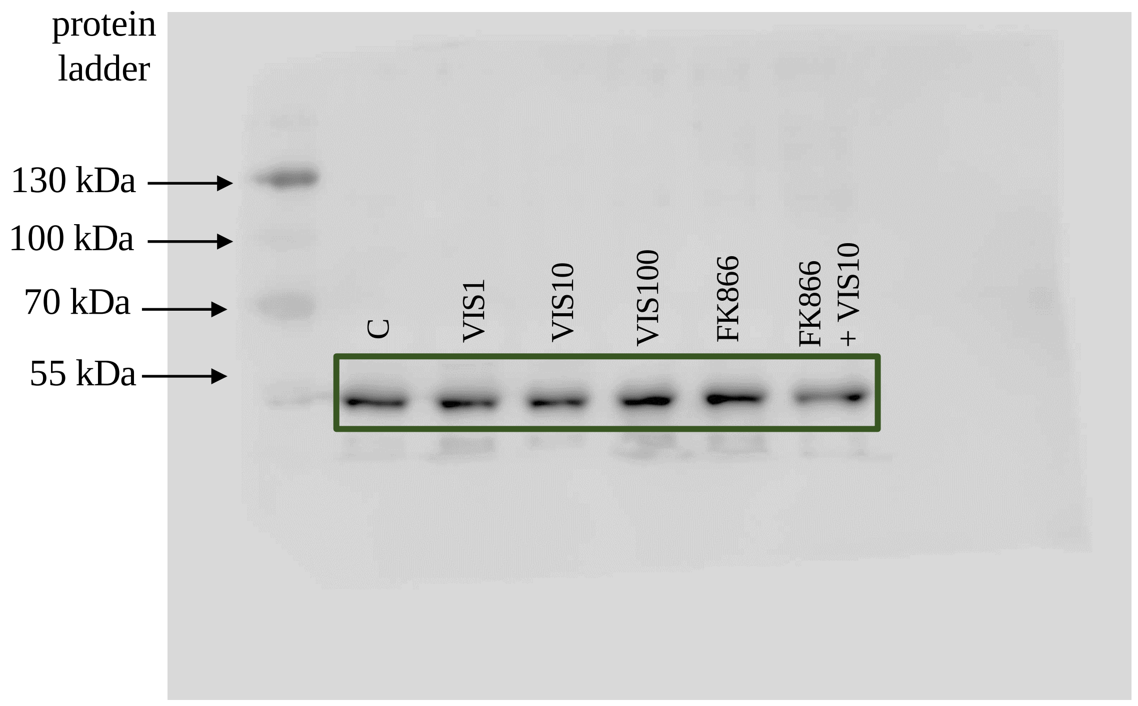

In vitro effect of visfatin on endocrine functions of the porcine corpus luteum

Ewa Mlyczyńska, Edyta Rytelewska, Ewa Zaobidna, Natalia Respekta, Grzegorz Kopij, Kamil Dobrzyń, Marta Kieżun, Nina Smolińska, Tadeusz Kamiński, Agnieszka Rak

Representative original blots for the expression of steroidogenic enzymes under the influence of visfatin included in **Figure 3H**.

CYP19A1

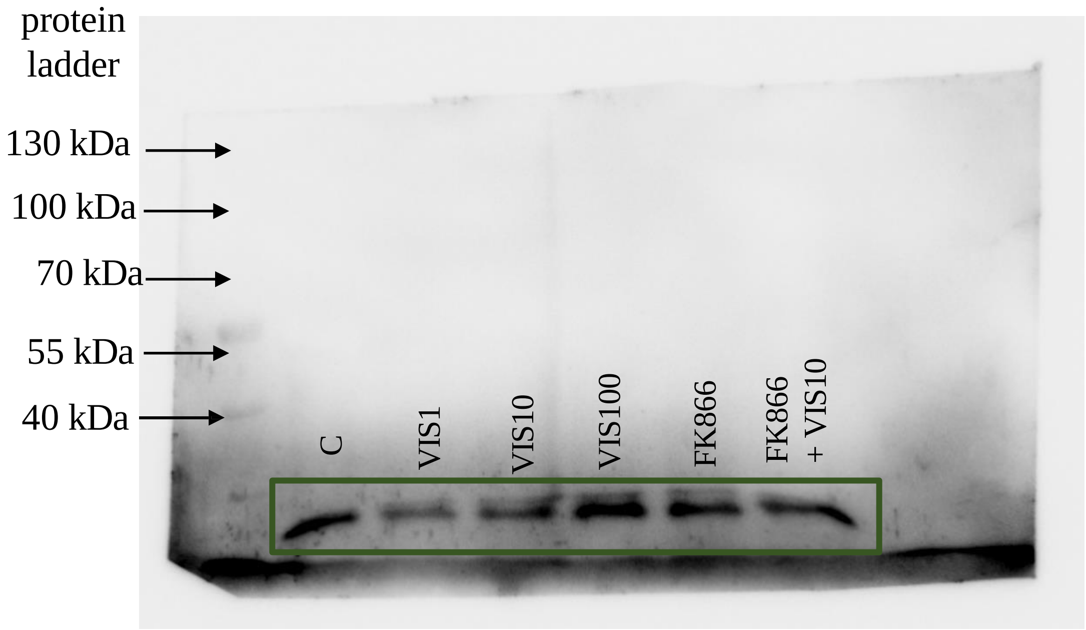

ACTIN

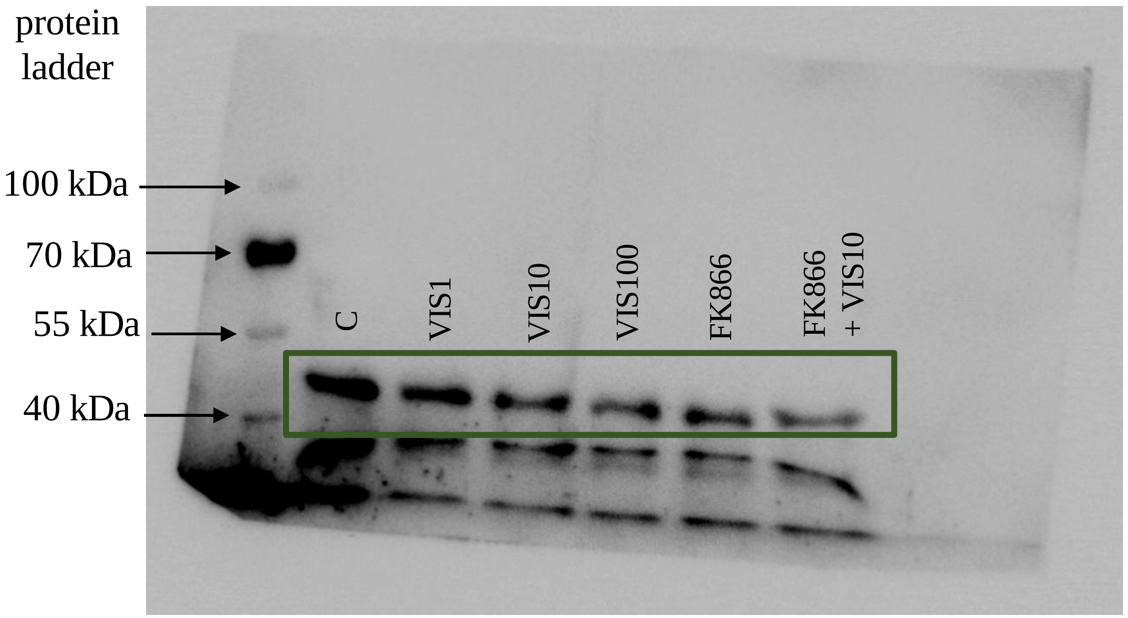

In vitro effect of visfatin on endocrine functions of the porcine corpus luteum

Ewa Mlyczyńska, Edyta Rytelewska, Ewa Zaobidna, Natalia Respekta, Grzegorz Kopij, Kamil Dobrzyń, Marta Kieżun, Nina Smolińska, Tadeusz Kamiński, Agnieszka Rak

Representative original blots for the expression of prostaglandins' receptors under the influence of visfatin included in **Figure 5B**.

PTGER2

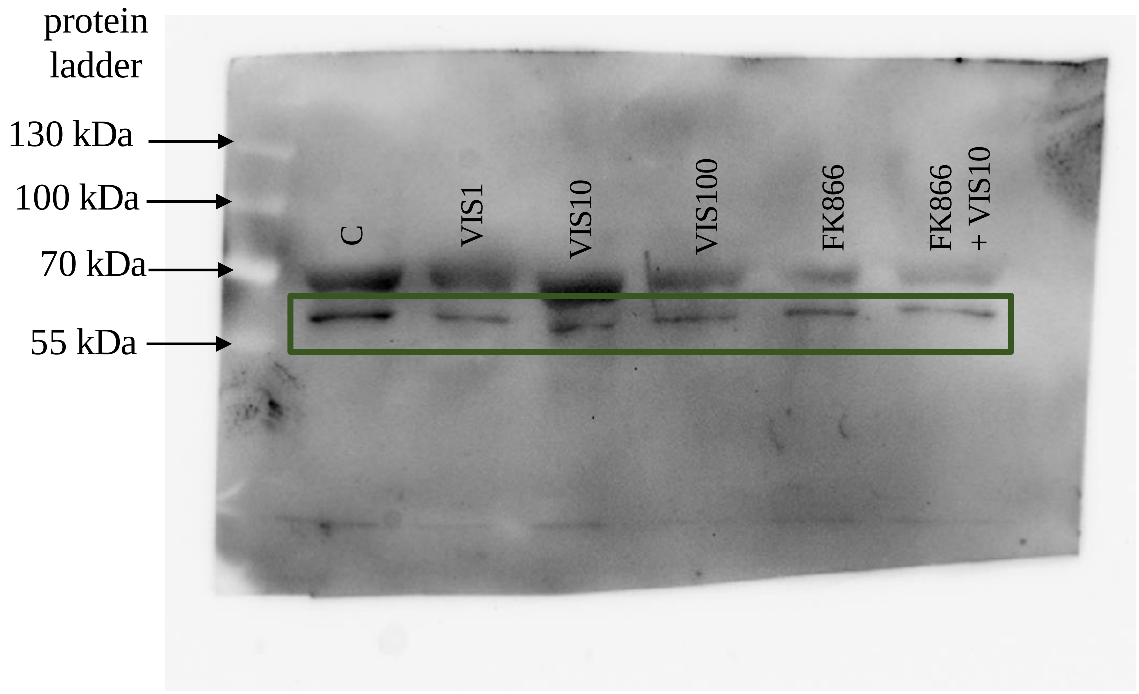

ACTIN

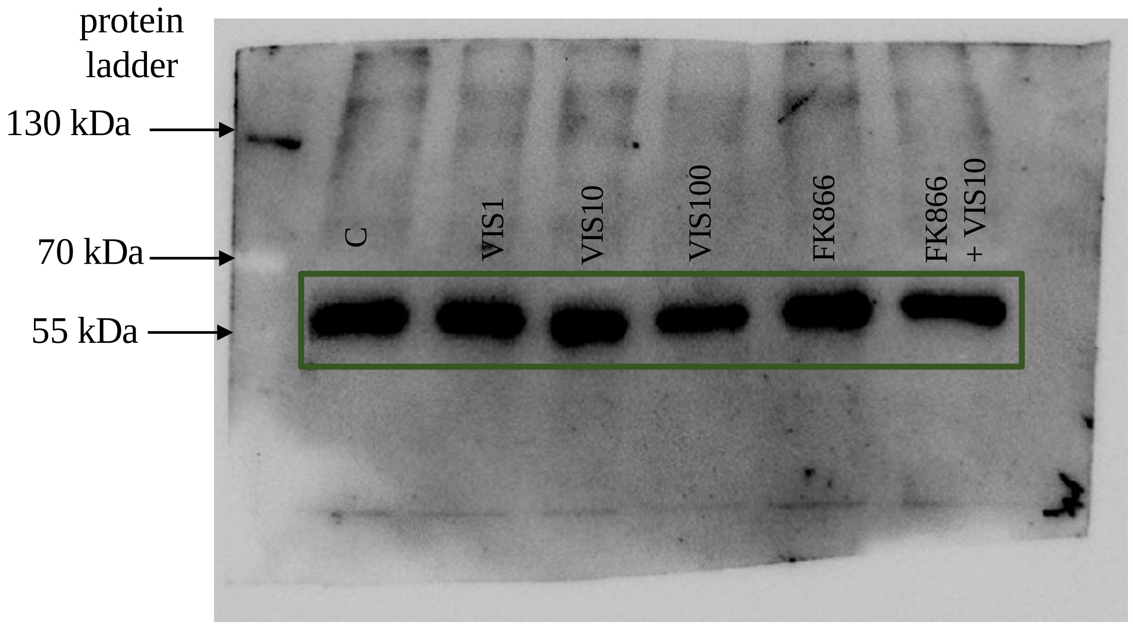

In vitro effect of visfatin on endocrine functions of the porcine corpus luteum

Ewa Mlyczyńska, Edyta Rytelewska, Ewa Zaobidna, Natalia Respekta, Grzegorz Kopij, Kamil Dobrzyń, Marta Kieżun, Nina Smolińska, Tadeusz Kamiński, Agnieszka Rak

Representative original blots for the expression of prostaglandins' receptors under the influence of visfatin included in **Figure 5D**.

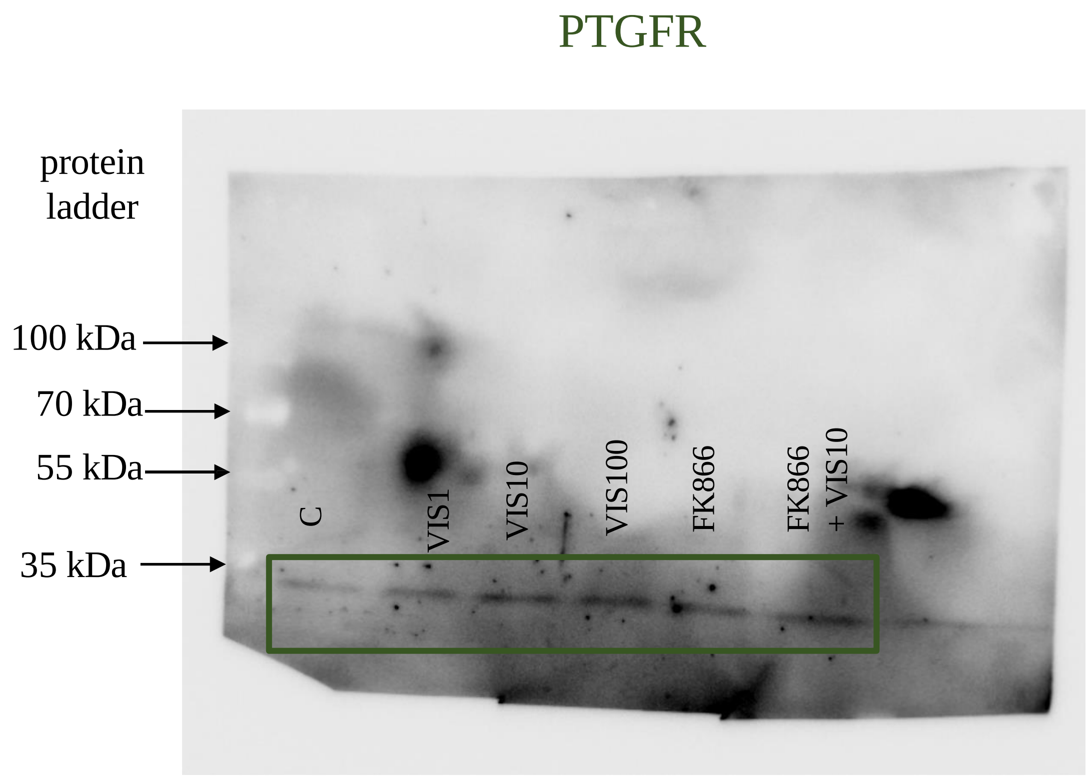

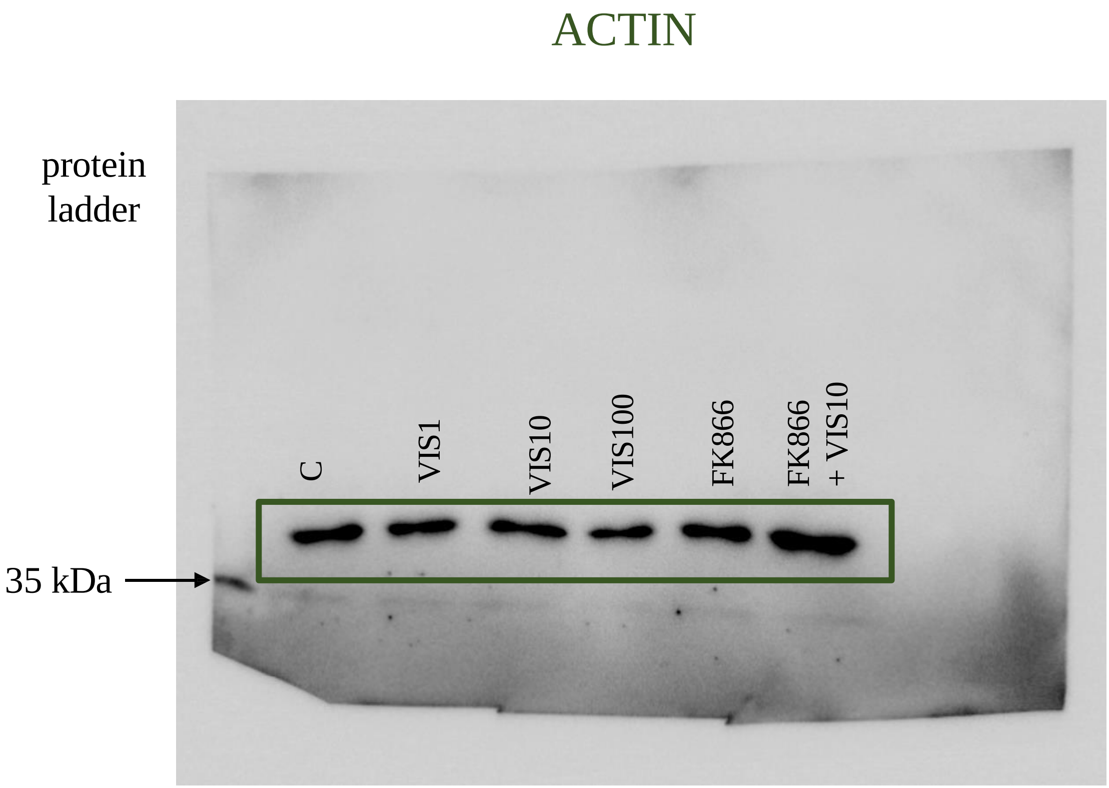

In vitro effect of visfatin on endocrine functions of the porcine corpus luteum

Ewa Mlyczyńska, Edyta Rytelewska, Ewa Zaobidna, Natalia Respekta, Grzegorz Kopij, Kamil Dobrzyń, Marta Kieżun, Nina Smolińska, Tadeusz Kamiński, Agnieszka Rak

Representative original blots for the expression of phosporylated and total form of kinases under the influence of visfatin included in **Figure 6B**.

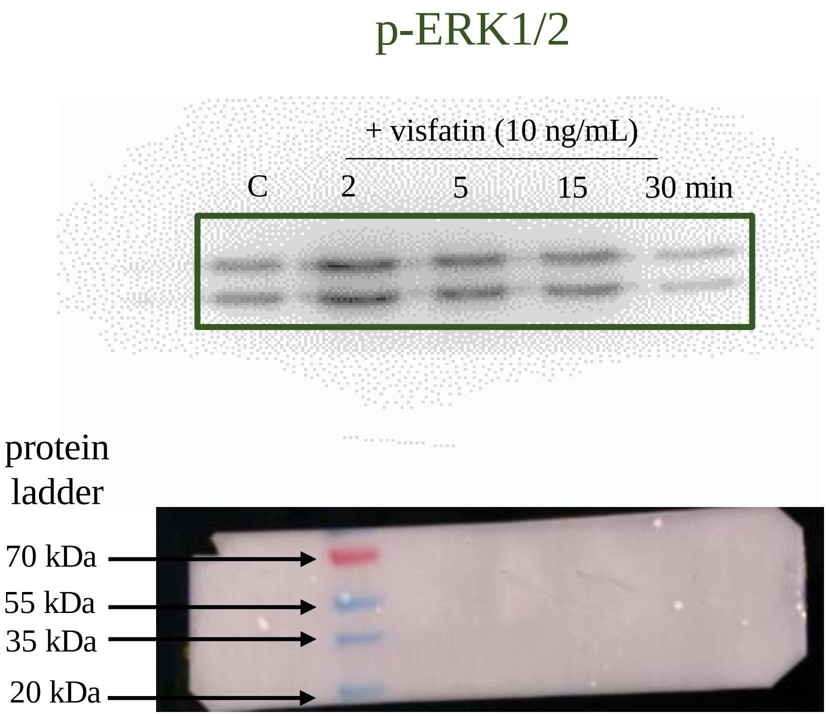

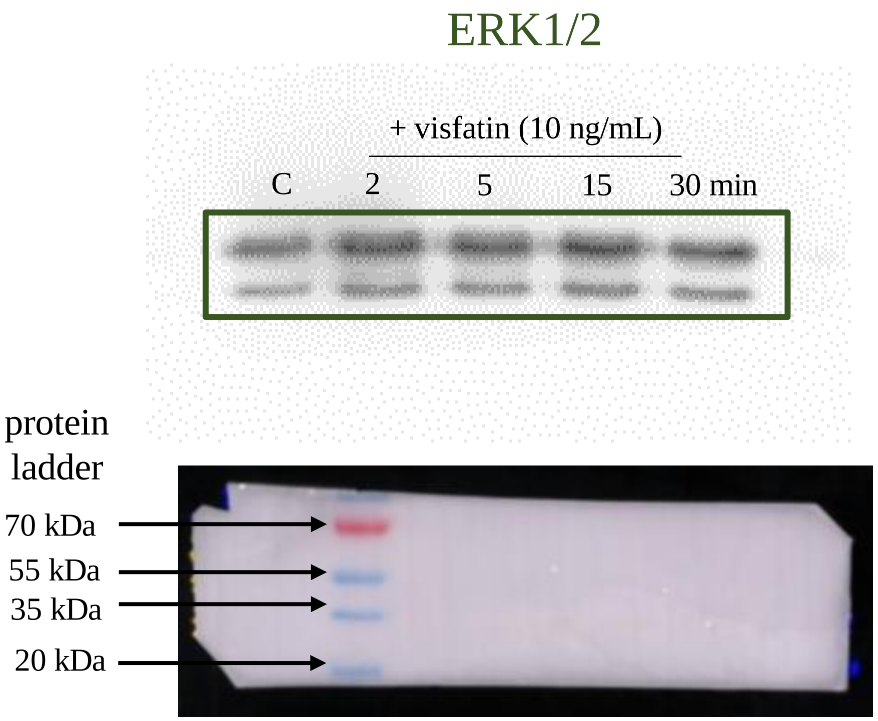

Blots were cut according to the molecular weights of the tested proteins and based on the protein ladder.

In vitro effect of visfatin on endocrine functions of the porcine corpus luteum

Ewa Mlyczyńska, Edyta Rytelewska, Ewa Zaobidna, Natalia Respekta, Grzegorz Kopij, Kamil Dobrzyń, Marta Kieżun, Nina Smolińska, Tadeusz Kamiński, Agnieszka Rak

Representative original blots for the expression of phosporylated and total form of kinases under the influence of visfatin included in **Figure 6C**.

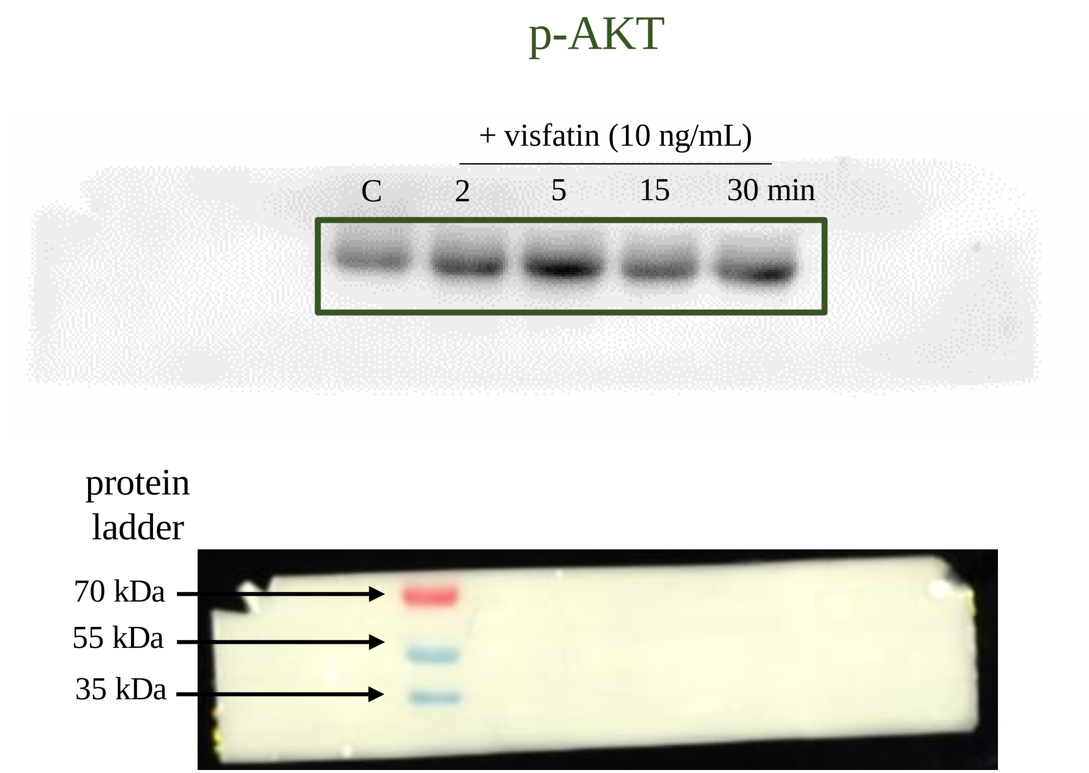

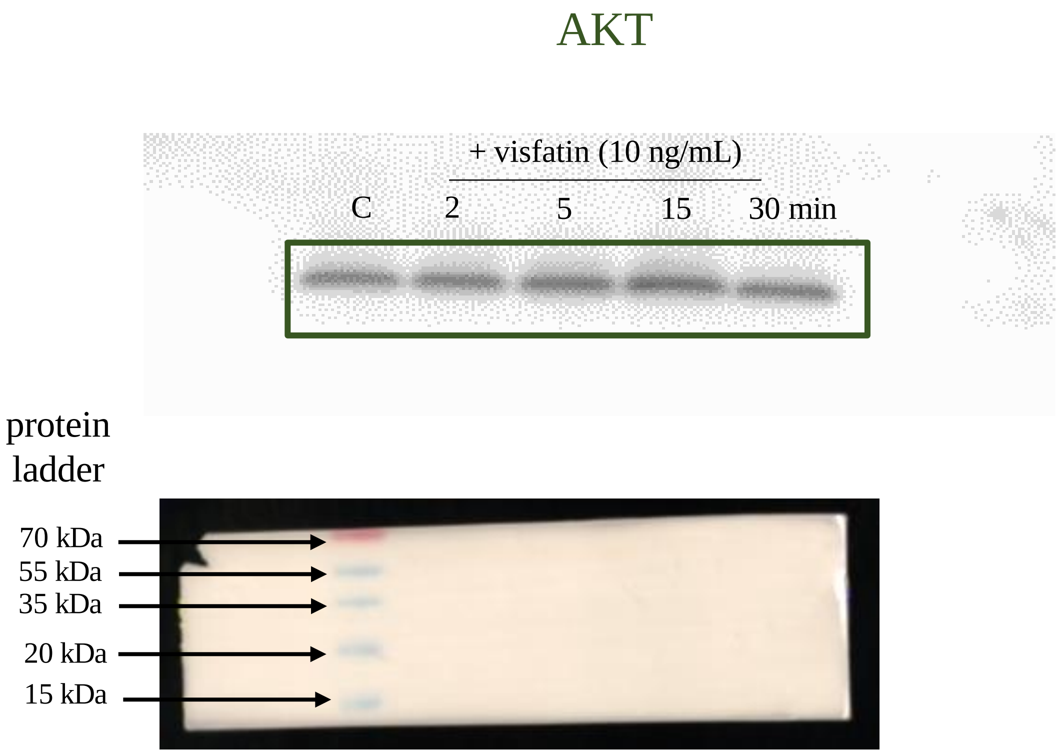

Blots were cut according to the molecular weights of the tested proteins and based on the protein ladder.

In vitro effect of visfatin on endocrine functions of the porcine corpus luteum

Ewa Mlyczyńska, Edyta Rytelewska, Ewa Zaobidna, Natalia Respekta, Grzegorz Kopij, Kamil Dobrzyń, Marta Kieżun, Nina Smolińska, Tadeusz Kamiński, Agnieszka Rak

Representative original blots for the expression of phosporylated and total form of kinases under the influence of visfatin included in **Figure 6D**.

p-AMPK

+ visfatin (10 ng/mL)

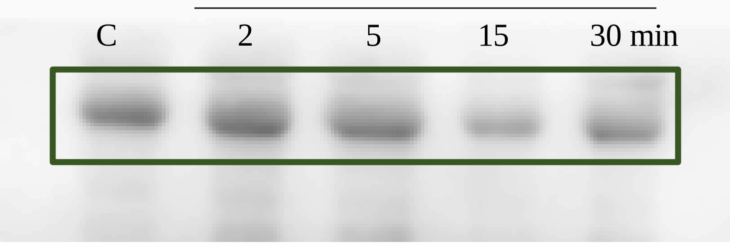

protein ladder

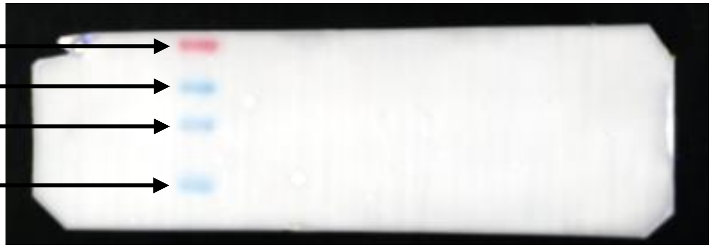

AMPK

+ visfatin (10 ng/mL)

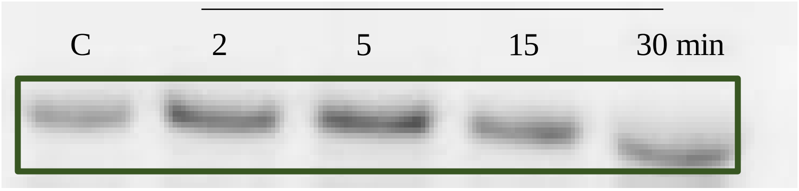

protein ladder

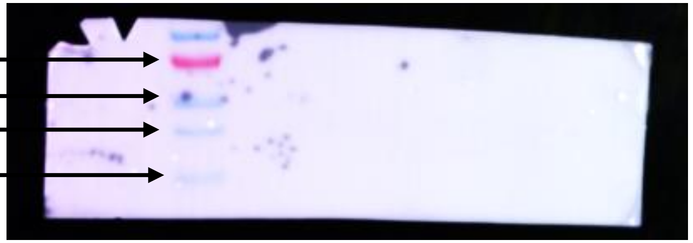

Blots were cut according to the molecular weights of the tested proteins and based on the protein ladder.
