# Supplementary material for: In vitro effect of visfatin on endocrine functions of the porcine corpus luteum
Source: Sci Rep. 2024 Jun 26;14:14780. doi: 10.1038/s41598-024-65102-4 (PMC11208563; doi:10.1038/s41598-024-65102-4)
Supplement: Supplementary file 2 — Supplementary Table 1. [file 41598_2024_65102_MOESM2_ESM.docx]

Supplementary Table 1. The main factors and the interactions of these factors affecting the

progesterone secretion by the porcine luteal cells during the estrous cycle (the results of two-way ANOVA).

|  | DAYS OF THE ESTROUS CYCLE | FACTORS | F | p |
| --- | --- | --- | --- | --- |
| basal secretion | 2 – 3 | VIS | F _(3, 29)_ = 4.137 | p= 0.0155 |
|  |  | FK866 | F _(1, 29)_ = 101.5 | p < 0.0001 |
|  |  | VIS*FK866 | F _(3, 29)_ = 38.58 | p < 0.0001 |
|  | 10 – 12 | VIS | F _(3, 29)_ = 10.31 | p < 0.0001 |
|  |  | FK866 | F _(1, 29)_ = 22.65 | p < 0.0001 |
|  |  | VIS*FK866 | F _(3, 29)_ = 9.029 | p = 0.0002 |
|  | 14 – 16 | VIS | F _(3, 29)_ = 22.80 | p < 0.0001 |
|  |  | FK866 | F _(1, 29)_ = 6.265 | p = 0.0182 |
|  |  | VIS*FK866 | F _(3, 29)_ = 6.026 | p = 0.0025 |
| LH-stimulated | 2 – 3 | VIS | F _(1, 14)_ = 25.67 | p = 0.0002 |
|  |  | FK866 | F _(1, 14)_ = 3.68 | p = 0.0771 |
|  |  | VIS*FK866 | F _(1, 14)_ = 0.244 | p = 0.6294 |
|  | 10 – 12 | VIS | F _(1, 14)_ = 3.631 | p = 0.0775 |
|  |  | FK866 | F _(1, 14)_ = 73.42 | p < 0.0001 |
|  |  | VIS*FK866 | F _(1, 14)_ = 50.99 | p < 0.0001 |
|  | 14 – 16 | VIS | F _(1, 14)_ = 27.79 | p = 0.0001 |
|  |  | FK866 | F _(1, 14)_ = 13.41 | p = 0.0026 |
|  |  | VIS*FK866 | F _(1, 14)_ = 19.19 | p = 0.0006 |
| INS-stimulated | 2 – 3 | VIS | F _(1, 14)_ = 21.62 | p = 0.0006 |
|  |  | FK866 | F _(1, 14)_ = 5.111 | p = 0.0432 |
|  |  | VIS*FK866 | F _(1, 14)_ = 2.010 | p = 0.1817 |
|  | 10 – 12 | VIS | F _(1, 16)_ = 4.709 | p = 0.0454 |
|  |  | FK866 | F _(1, 16)_ = 1.397 | p = 0.2545 |
|  |  | VIS*FK866 | F _(1, 16)_ = 0.032 | p = 0.8602 |
|  | 14 – 16 | VIS | F _(1, 14)_ = 20.97 | p = 0.0005 |
|  |  | FK866 | F _(1, 14)_ = 0.133 | p = 0.4773 |
|  |  | VIS*FK866 | F _(1, 14)_ = 0.535 | p = 0.7213 |
| LH + INS-stimulated | 2 – 3 | VIS | F (_1, 16)_ = 14.95 | p = 0.0014 |
|  |  | FK866 | F _(1, 16)_ = 4.629 | p = 0.0470 |
|  |  | VIS*FK866 | F _(1, 16)_ = 9.424 | p = 0.0073 |
|  | 10 – 12 | VIS | F _(1, 14)_ = 13.91 | p = 0.0025 |
|  |  | FK866 | F _(1, 14)_ = 2.783 | p = 0.1192 |
|  |  | VIS*FK866 | F _(1, 14)_ = 2.639 | p = 0.1282 |
|  | 14 – 16 | VIS | F _(1, 14)_ = 43.58 | p < 0.0001 |
|  |  | FK866 | F _(1, 14)_ = 47.13 | p < 0.0001 |
|  |  | VIS*FK866 | F _(1, 14)_ = 27.75 | p = 0.0001 |
