# Supplementary material for: In vitro effect of visfatin on endocrine functions of the porcine corpus luteum
Source: Sci Rep. 2024 Jun 26;14:14780. doi: 10.1038/s41598-024-65102-4 (PMC11208563; doi:10.1038/s41598-024-65102-4)
Supplement: Supplementary file 3 — Supplementary Table 2. [file 41598_2024_65102_MOESM3_ESM.docx]

Supplementary Table 2. The main factors and the interactions of these factors affecting the

estradiol secretion by the porcine luteal cells during the estrous cycle (the results of two-way ANOVA).

|  | DAYS OF THE ESTROUS CYCLE | FACTORS | F | p |
| --- | --- | --- | --- | --- |
| basal secretion | 2 – 3 | VIS | F _(3, 31)_ = 4.418 | p = 0.0107 |
|  |  | FK866 | F _(1, 31)_ = 0.0615 | p = 0.8057 |
|  |  | VIS*FK866 | F _(3, 31)_ = 1.031 | p = 0.3923 |
|  | 10 – 12 | VIS | F _(3, 32)_ = 21.07 | p < 0.0001 |
|  |  | FK866 | F _(1, 32)_ = 131.8 | p < 0.0001 |
|  |  | VIS*FK866 | F _(3, 32)_ = 16.79 | p < 0.0001 |
|  | 14 – 16 | VIS | F _(3, 31)_ = 67.45 | p < 0.0001 |
|  |  | FK866 | F _(1, 31)_ = 149.6 | p < 0.0001 |
|  |  | VIS*FK866 | F _(3, 31)_ = 57.32 | p < 0.0001 |
| LH-stimulated | 2 – 3 | VIS | F _(1, 15)_ = 6.944 | p = 0.0187 |
|  |  | FK866 | F _(1, 15)_ = 24.00 | p = 0.0002 |
|  |  | VIS*FK866 | F _(1, 15)_ = 7.066 | p = 0.0179 |
|  | 10 – 12 | VIS | F _(1, 14)_ = 0.096 | p = 0.7612 |
|  |  | FK866 | F _(1, 14)_ = 0.012 | p = 0.9128 |
|  |  | VIS*FK866 | F _(1, 14)_ = 3.544 | p = 0.0807 |
|  | 14 – 16 | VIS | F _(1, 15)_ = 30.40 | p < 0.0001 |
|  |  | FK866 | F _(1, 15)_ = 6.749 | p = 0.0202 |
|  |  | VIS*FK866 | F _(1, 15)_ = 23.65 | p = 0.0002 |
| INS-stimulated | 2 – 3 | VIS | F _(1, 13)_ = 31.50 | p < 0.0001 |
|  |  | FK866 | F _(1, 13)_ = 62.10 | p < 0.0001 |
|  |  | VIS*FK866 | F _(1, 13)_ = 1.844 | p = 0.1976 |
|  | 10 – 12 | VIS | F _(1, 14)_ = 1.259 | p = 0.2808 |
|  |  | FK866 | F _(1, 14)_ = 1.255 | p = 0.2814 |
|  |  | VIS*FK866 | F _(1, 14)_ = 0.1149 | p = 0.7397 |
|  | 14 – 16 | VIS | F _(1, 14)_ = 75.89 | p < 0.0001 |
|  |  | FK866 | F _(1, 14)_ = 0.6943 | p = 0.4187 |
|  |  | VIS*FK866 | F _(1, 14)_ = 0.1065 | p = 0.7490 |
| LH + INS-stimulated | 2 – 3 | VIS | F _(1, 14)_ = 6.450 | p = 0,0236 |
|  |  | FK866 | F _(1, 14)_ = 15.32 | p = 0.0016 |
|  |  | VIS*FK866 | F _(1, 14)_ = 0.2667 | p = 0.6136 |
|  | 10 – 12 | VIS | F _(1, 14)_ = 3.392 | p = 0.0868 |
|  |  | FK866 | F _(1, 14)_ = 1.489 | p = 0.2426 |
|  |  | VIS*FK866 | F _(1, 14)_ = 0.7096 | p = 0.4137 |
|  | 14 – 16 | VIS | F _(1, 14)_ = 8.112 | p = 0.0129 |
|  |  | FK866 | F _(1, 14)_ = 0.0107 | p = 0.9192 |
|  |  | VIS*FK866 | F _(1, 14)_ = 10.41 | p = 0.0061 |
