# Supplementary material for: In vitro effect of visfatin on endocrine functions of the porcine corpus luteum
Source: Sci Rep. 2024 Jun 26;14:14780. doi: 10.1038/s41598-024-65102-4 (PMC11208563; doi:10.1038/s41598-024-65102-4)
Supplement: Supplementary file 4 — Supplementary Table 3. [file 41598_2024_65102_MOESM4_ESM.docx]

Supplementary Table 3: Experimental setup

|  | Days of the estrous cycle | Treatment | Time of incubation | Parameters | Methods |
| --- | --- | --- | --- | --- | --- |
| Experiment 1 | 2–3, 10–12, 14–16 | -Visfatin (1, 10, 100 ng/mL)  -LH (100 ng/mL) alone or with visfatin (10 ng/mL)  -INS (10 ng/mL) alone or with visfatin (10 ng/mL)  -LH (100 ng/mL) +INS (10 ng/mL) alone or with visfatin (10 ng/mL)  -FK866 (10 nM) alone or with visfatin (1, 10, 100 ng/mL)  -LH (100 ng/mL) alone or with visfatin (10 ng/mL) + FK866 (10 nM)  -INS (10 ng/mL) alone or with visfatin (10 ng/mL) + FK866 (10 nM)  -LH (100 ng/mL) + INS (10 ng/mL) alone or with visfatin  (10 ng/mL) + FK866 (10 nM) | 24 h | P_4_ concentration | RIA assay |
|  |  |  |  | E_2_ concentration | ELISA assay |
| Experiment 2 | 10–12 | -Visfatin (1, 10, 100 ng/mL)  -FK866 (10 nM) alone or with visfatin (10 ng/mL) |  | STAR, CYP11A1, HSD3B, CYP19A1 transcript/protein level | real-time PCR/ western blot |
| Experiment 3 | 2–3, 10–12, 14–16 | -Visfatin (1, 10, 100 ng/mL)  -FK866 (10 nM) alone or with visfatin (10 ng/mL) |  | PGE_2_ and PGF_2α_ concentration | ELISA assay |
| Experiment 4 | 10–12 | -Visfatin (1, 10, 100 ng/mL)  -FK866 (10 nM) alone or with visfatin (10 ng/mL) |  | PTGER2 transcript/protein level | real-time PCR/ western blot |
|  | 14–16 |  |  | PTGFR transcript/protein level | real-time PCR/ western blot |
| Experiment 5 | 10–12 | -Visfatin (10 ng/mL) | 2, 5, 10, 30 min | p-INSR and INSR concentration | ELISA assay |
|  |  |  |  | p-ERK1/2, ERK1/2  p-AKT, AKT  p-AMPK, AMPK | western blot |
| Experiment 6 | 10–12 | -S961 (1 µM) or LY294002 (20 µM) or U0126 (10 µM) or Dorsomorphin (10 µM) alone or with visfatin (10 ng/mL) | 24 h | P_4_ concentration | RIA assay |
|  |  |  |  | E_2_ concentration | ELISA assay |
|  | 10–12 | -S961 (1 µM) or U0126 (10 µM) alone or with visfatin  (10 ng/mL) |  | PGE_2_ concentration | ELISA assay |
|  | 14–16 |  |  | PGF_2α_ concentration | ELISA assay |

**Abbreviations:** FK866 – selective blocker of enzymatic activity of visfatin, LH – luteinizing hormone, INS – insulin, P_4_ – progesterone, E_2_ – estradiol, STAR – steroidogenic acute regulatory protein, CYP11A1 – cytochrome P450 family 11 subfamily A member 1, HSD3B – hydroxy-delta-5-steroid dehydrogenase, CYP19A1 – cytochrome P450 family 19 subfamily A member 1, PGE_2_ – prostaglandin E_2_, PGF_2α_ – prostaglandin F_2α_, PTGER2 – receptor of PGE_2_, PTGFR – receptor of PGF_2α_, p – phosphorylated, INSR – insulin receptor, ERK1/2 – extracellular signal-regulated kinase 1/2, AKT – protein kinase B, AMPK – 5'AMP-activated protein kinase, S961 – blocker of INSR pathway, LY294002 – blocker of AKT pathway, U0126 – blocker of MAPK/ERK1/2 pathway, Dorsomorphin – blocker of AMPK pathway.
