# Supplementary material for: In vitro effect of visfatin on endocrine functions of the porcine corpus luteum
Source: Sci Rep. 2024 Jun 26;14:14780. doi: 10.1038/s41598-024-65102-4 (PMC11208563; doi:10.1038/s41598-024-65102-4)
Supplement: Supplementary file 5 — Supplementary Table 4. [file 41598_2024_65102_MOESM5_ESM.docx]

Supplementary Table 4. Characteristic of ELISA assays used in study.

| **Target protein** | **Cat. no. and supplier** | **Standard curve range** | **Sensitivity of the assay** | **Volume of sample** | **Intra-assay coefficient of variation** | **Inter-assay coefficient of variation** |
| --- | --- | --- | --- | --- | --- | --- |
| **Estradiol** | EIA-2693, DRG Instruments GmbH, Marburg, Germany | 10.6 – 2000 pg/mL | 10.6 pg/mL | 25 µL | <10% | <15% |
| **Prostaglandin E_2_** | EP0205, FineTest, Nanjing, China | 31.25 – 2000 pg/mL | 18.75 pg/mL | 50 µL | <8% | <10% |
| **Prostaglandin F_2α_** | EP0206, FineTest, Nanjing, China | 7.81 – 500 pg/mL | 4.68 pg/mL | 50 µL | <8% | <10% |
| **Phospho-Insulin receptor** | EIA09483p, Enlibio Biotech Co., Wuhan, China | 0.156 – 10 ng/mL | 0.05 ng/mL | 100 µL | <8% | does not apply |
| **Total-Insulin receptor** | EIA05929p, Enlibio Biotech Co., Wuhan, China | 0.312 – 20 ng/mL | 0.06 ng/mL | 100 µL | <8% | does not apply |
