# Supplementary material for: In vitro effect of visfatin on endocrine functions of the porcine corpus luteum
Source: Sci Rep. 2024 Jun 26;14:14780. doi: 10.1038/s41598-024-65102-4 (PMC11208563; doi:10.1038/s41598-024-65102-4)
Supplement: Supplementary file 6 — Supplementary Table 5. [file 41598_2024_65102_MOESM6_ESM.doc]

Supplementary Table 5. Specifications of antibodies used for western blot analysis.

| **Antibody** | **Species of protein used to generate antibody** | | | **Host** | **Dilution** | **Cat. No.** | **Supplier** |
| --- | --- | --- | --- | --- | --- | --- | --- |
| STAR | human | | | rabbit | 1:500 | ab233427 | Abcam, Cambridge, UK |
| CYP11A1 | human | | | rabbit | 1:1000 | ab175408 | Abcam, Cambridge, UK |
| HSD3B | human | | | mouse | 1:1000 | ab75710 | Abcam, Cambridge, UK |
| CYP19A1 | human | | | rabbit | 1:200 | PA1-21398 | Thermo Fisher Scientific,  MA, USA |
| PTGER2 | | human | | rabbit | 1:200 | PA5-91872 | Thermo Fisher Scientific,  MA, USA |
| PTGFR | | human | | rabbit | 1:500 | PA5-70674 | Thermo Fisher Scientific,  MA, USA |
| phospho-ERK1/2 | | chicken | | rabbit | 1:1000 | #9101S | Cell Signaling Technology,  MA, USA |
| ERK1/2 | | rat | | rabbit | 1:1000 | #9102S | Cell Signaling Technology,  MA, USA |
| phospho-AKT | | | mouse | rabbit | 1:1000 | #9271S | Cell Signaling Technology,  MA, USA |
| AKT | | | mouse | rabbit | 1:1000 | #9272S | Cell Signaling Technology,  MA, USA |
| phospho-AMPK | | | human | rabbit | 1:1000 | #2531S | Cell Signaling Technology,  MA, USA |
| AMPK | | | human | rabbit | 1:1000 | #2532S | Cell Signaling Technology,  MA, USA |
| actin | | | human | mouse | 1:1000 | A5316 | Sigma-Aldrich, MO, USA |
| anti-rabbit | | | does not apply | goat | 1:1000 | #7074 | Cell Signaling Technology,  MA, USA |
| anti-mouse | | | does not apply | horse | 1:1000 | #7076 | Cell Signaling Technology,  MA, USA |
