# Supplementary material for: In vitro effect of visfatin on endocrine functions of the porcine corpus luteum
Source: Sci Rep. 2024 Jun 26;14:14780. doi: 10.1038/s41598-024-65102-4 (PMC11208563; doi:10.1038/s41598-024-65102-4)
Supplement: Supplementary file 7 — Supplementary Table 6. [file 41598_2024_65102_MOESM7_ESM.docx]

Supplementary Table 6. The F-value and the corresponding p-value obtained in the ANOVA test.

| Figure no. | Panel | F value | p value |
| --- | --- | --- | --- |
| Figure 1 | A | F _(19, 68)_ = 21.78 | p < 0.0001 |
|  | B | F _(19, 72)_ = 16.10 | p < 0.0001 |
|  | C | F _(19, 71_) = 29.31 | p < 0.0001 |
| Figure 2 | A | F _(19, 70)_ = 20.03 | p < 0.0001 |
|  | B | F _(19, 68)_ = 15.28 | p < 0.0001 |
|  | C | F _(19, 72)_ = 26.58 | p < 0.0001 |
| Figure 3 | A | F _(5, 17)_ = 2.199 | p =0.0134 |
|  | B | F _(5, 24)_ = 207.3 | p < 0.0001 |
|  | C | F _(5, 24)_ = 6.406 | p = 0.0007 |
|  | D | F _(5, 24)_ = 401.8 | p < 0.0001 |
|  | E | F _(5, 31_) = 27.90 | p < 0.0001 |
|  | F | F _(5, 24)_ = 279.1 | p < 0.0001 |
|  | G | F _(5, 17)_ = 13.84 | p < 0.0001 |
|  | H | F _(5, 12)_ = 779.9 | p < 0.0001 |
| Figure 4 | A | F _(5, 23)_ = 17.46 | p = 0.0585 |
|  | B | F _(5, 26)_ = 35.05 | p < 0.0001 |
|  | C | F _(5, 24)_ = 3.495 | p = 0.0163 |
|  | D | F _(5, 25)_ = 27.72 | p < 0.0001 |
|  | E | F _(5, 26)_ = 1.367 | p = 0.2688 |
|  | F | F _(5, 20)_ = 1.493 | p < 0.0001 |
| Figure 5 | A | F _(5, 13)_ = 7.307 | p = 0.0018 |
|  | B | F _(5, 12)_ = 274.9 | p < 0.0001 |
|  | C | F _(5, 26)_ = 4.783 | p = 0.0031 |
|  | D | F _(5, 12)_ = 316.8 | p < 0.0001 |
| Figure 6 | A | F _(4, 20)_ = 10.29 | p = 0.0001 |
|  | B | F _(4, 18)_ = 17,83 | p < 0.0001 |
|  | C | F _(4, 20)_ = 43.97 | p < 0.0001 |
|  | D | F _(4, 19)_ = 38.48 | p < 0.0001 |
| Figure 7 | A | F _(9, 39_) = 22.39 | p < 0.0001 |
|  | B | F _(9, 37)_ = 2.034 | p = 0.0130 |
|  | C | F _(5, 24)_ = 6.884 | p = 0.0004 |
|  | D | F _(5, 24)_ = 5.709 | p = 0.0013 |
